# Competitive displacement of lipoprotein lipase from heparan sulfate is orchestrated by a disordered acidic cluster in GPIHBP1

**DOI:** 10.1016/j.jlr.2025.100745

**Published:** 2025-01-13

**Authors:** Anamika Biswas, Samina Arshid, Kristian Kølby Kristensen, Thomas J.D. Jørgensen, Michael Ploug

**Affiliations:** 1 Finsen Laboratory, Copenhagen University Hospital, Rigshospitalet, Copenhagen, Denmark; 2 Biotech Research and Innovation Centre, University of Copenhagen, Copenhagen, Denmark; 3 Department of Biochemistry and Molecular Biology, University of Southern Denmark, Odense, Denmark

**Keywords:** lipase/lipoprotein, transport, lipolysis and fatty acid metabolism, triglycerides, ANGPTL4, intrinsically disordered regions, PSCK3 cleavage, competitive displacement, polyelectrolytes

## Abstract

Movement of lipoprotein lipase (LPL) from myocytes or adipocytes to the capillary lumen is essential for intravascular lipolysis and plasma triglyceride homeostasis—low LPL activity in the capillary lumen causes hypertriglyceridemia. The *trans*-endothelial transport of LPL depends on ionic interactions with GPIHBP1’s intrinsically disordered N-terminal tail, which harbors two acidic clusters at positions 5–12 and 19–30. This polyanionic tail provides a molecular switch that controls LPL detachment from heparan sulfate proteoglycans (HSPGs) by competitive displacement. When the acidic tail was neutralized in gene-edited mice, LPL remained trapped in the sub-endothelial spaces triggering hypertriglyceridemia. Due to its disordered state, the crystal structure of LPL•GPIHBP1 provided no information on these electrostatic interactions between LPL and GPIHBP1 acidic tail. In the current study, we positioned the acidic tail on LPL using zero-length crosslinking. Acidic residues at positions 19–30 in GPIHBP1 mapped to Lys^445^, Lys^441^, Lys^414^, and Lys^407^ close to the interface between the C- and N-terminal domains in LPL. Modeling this interface revealed widespread polyelectrolyte interactions spanning both LPL domains, which explains why the acidic tail stabilizes LPL activity and protein conformation. In functional assays, we showed that the acidic cluster at 19–30 also had the greatest impact on preserving LPL activity, mitigating ANGPTL4-catalyzed LPL inactivation, preventing PSCK3-mediated LPL cleavage, and, importantly, displacing LPL from HSPGs. Our current study provides key insights into the biophysical mechanism(s) orchestrating intravascular compartmentalization of LPL activity—an intriguing pathway entailing competitive displacement of HSPG-bound LPL by a disordered acidic tail in GPIHBP1.

Genetic and epidemiologic evidence support a causal role of elevated plasma levels of triglyceride-rich lipoproteins (TRLs) and their cholesterol-enriched remnant particles in the risk of acute pancreatitis and atherosclerotic cardiovascular disease (ASCVD) (1, 2, 3, 4, 5). The rate-limiting enzyme hydrolyzing plasma triglycerides into free fatty acids and converting TRLs (chylomicrons or VLDL) into their corresponding remnant particles is lipoprotein lipase (LPL). Rapid conversion of TRLs into remnants thus depends on a highly regulated flux of active LPL from its site of synthesis in myocytes or adipocytes to its site of action on the luminal membrane of capillary endothelial cells. The physiological relevance of this LPL flux is strongly supported by human genetics. Bi-allelic loss-of-function variants in *LPL* or in other genes promoting LPL activity in the capillary lumen (e.g., *APOC2, APOA5, LMF1, GPIHBP1*) cause severe hypertriglyceridemia with a predisposition to acute pancreatitis (6, 7). Disabling *trans*-capillary LPL transport by inhibitory GPIHBP1 autoantibodies also causes hypertriglyceridemia (8, 9). In contrast, individuals with bi-allelic loss-of-function variants in genes with a negative impact on LPL activity (e.g., *ANGPTL3, ANGPTL4, ANGPTL8, APOC3*) have reduced plasma TRL levels and are at reduced risk for ASCVD (10, 11, 12, 13). To satisfy distinct energy requirements of various tissues in fed and fasted states, LPL activity in the capillary lumen is tightly regulated in a tissue-specific manner by its inhibitors, angiopoietin-like (ANGPTL) proteins 3, 4, and 8 (14, 15, 16).

Early studies found that the lipase activity of purified LPL declined rapidly at 25°C (17) and recent biophysical studies revealed that this loss in LPL activity resulted from spontaneous unfolding of LPL’s α/β-hydrolase domain (18). This raises an important issue: How can a metastable enzyme like LPL function as the main workhorse for intravascular lipolysis? This conundrum was partly solved by subsequent studies using hydrogen–deuterium exchange mass spectrometry (HDX-MS). With this technology, we showed that the metastable nature of LPL plays a key role in the regulation of LPL activity by auxiliary proteins such as ANGPTL4, ANGPTL3/8, APOC2, and GPIHBP1 (19, 20). Irreversible inhibition of LPL activity by ANGPTL4 or ANGPTL3/8 is executed through a faster unfolding of LPL’s α/β-hydrolase domain i.e., ANGPTL-binding per se lowers the activation barrier of LPL unfolding (21, 22). Of note, ANGPTL4 and ANGPTL3/8 *catalyze* the inactivation of LPL ( 23) by destabilizing regions that anchor the lid domain to the α/β-hydrolase domain (22). In contrast, APOC2 acts as an activator of LPL-mediated TRL hydrolysis (24), but unexpectedly the APOC2 binding site on LPL overlaps with that of the inhibitor ANGPTL4 (20). Despite their shared binding sites on LPL, interactions with APOC2 elicit different allosteric effects on the conformation of the α/β-hydrolase domain. While ANGPTL4 and ANGPTL3/8 destabilize the lid-anchoring sequences in the α/β-hydrolase domain, APOC2 stabilizes the same region—well aligned with their distinct roles as an inhibitor and activator of LPL activity (20).

The focus of the current study is on GPIHBP1—the obligate endothelial trafficking receptor that shuttles interstitial LPL from its heparan-sulfate proteoglycan (HSPG) bound state to the luminal surface of capillary endothelial cells (25). GPIHBP1 is an atypical member of the Ly6/uPAR (LU) protein domain family (26) inasmuch its prototypical LU domain carries a unique 42-residue long N-terminal extension that is largely disordered and contains two acidic clusters flanking a sulfated tyrosine residue—as depicted in Fig. 1A, B (18, 28). This unique architecture shapes GPIHBP1 as a “functional and structural dipole”. At one pole, the folded LU domain provides a stable binding interface with LPL and an attachment site to the cell surface via a glycolipid anchor (Fig. 1C, D). At the opposite pole, the intrinsically disordered and acidic N-terminal tail creates an electrostatic “trap” (28, 29). In the crystal structure of the LPL•GPIHBP1 complex (27), this negatively charged extension of GPIHBP1 remained disordered. However, its position close to the heparin-binding motifs in LPL (Fig. 1C) suggested that extensive ionic interactions play a key role in the regulation and compartmentalization of LPL activity (19, 30). This is indeed the case. First, the acidic tail in GPIHBP1 enhances the association rate constant (*k*_*on*_) for LPL binding by 2,500-fold (28). Second, it protects monomeric LPL from inactivation via spontaneous or ANGPTL-catalyzed unfolding (18, 19, 21, 22, 31, 32). Third, GPIHBP1 mitigates LPL inactivation by ANGPTL4-induced PSCK3 cleavage (33, 34, 35) (Fig. 1D). Finally, the acidic tail of GPIHBP1 provides an electrostatic switch, which facilitates the dislodgment of LPL from its HSPG-bound state in the interstitial spaces. The detached LPL•GPIHBP1 complex is now available for subsequent trafficking to the capillary lumen (28). When the electrostatic switch was disabled in vivo by replacing the disordered acidic tail in GPIHBP1 with a neutral tail, these gene-edited mice became hypertriglyceridemic (29). This condition developed because LPL was trapped within the sub-endothelial spaces where it remained bound to interstitial HSPGs (29). In the current study, we clarified the molecular mechanisms by which GPIHBP1 dislodges LPL from its confinement on HSPGs. With zero-length crosslinking and molecular modeling, we defined the preferred binding ensemble for the intrinsically disordered acidic tail in GPIHBP1 on the basic heparin-binding surface of LPL. Furthermore, we measured the relative importance of two N-terminal acidic clusters on GPIHBP1 function. A key finding was that the conserved acidic charge-cluster 2 in GPIHBP1's intrinsically disordered tail is indispensable for the competitive displacement of HSPG-bound LPL and thus is expected to control *trans*-endothelial movement of LPL into the capillary lumen.

**Fig. 1:**
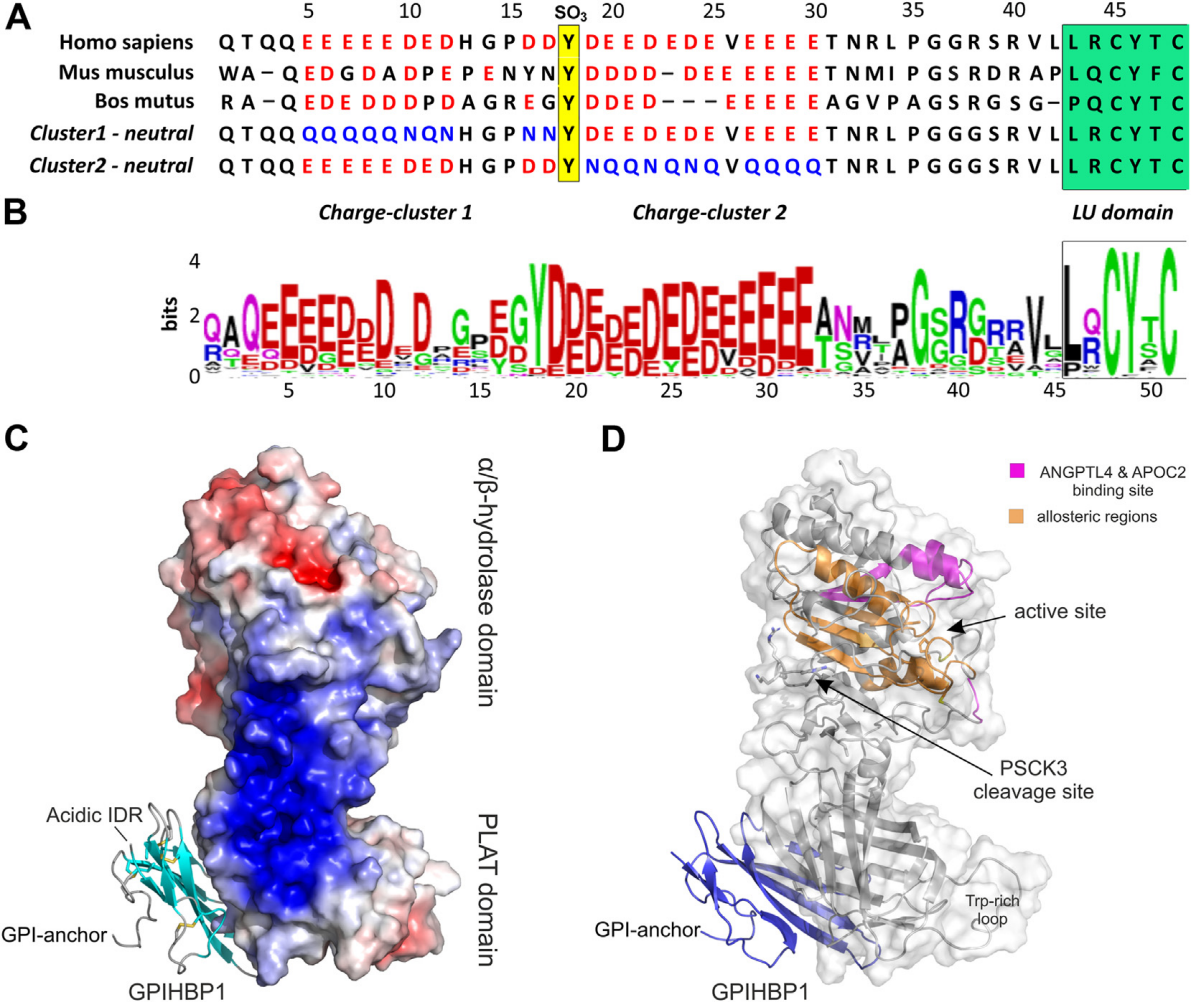
Overview of the LPL•GPIHBP1 complex. A: Sequence alignment of the disordered tail in human, mouse, and bovine GPIHBP1 with acidic residues highlighted in red and the sulfated tyrosine in yellow. Changes made in GPIHBP1 cluster 1 and 2 mutants are shown in blue. The start of the LU domain is highlighted by the green box. B: Sequence logo for the intrinsically disordered acidic extension of GPIHBP1 based on sequence alignments of GPIHBP1 from 45 mammalian species. C: Crystal structure of the human LPL•GPIHBP1 complex (27). GPIHBP1 is shown in a cartoon representation and LPL in an electrostatic surface representation. A coherent cationic surface including several of LPL’s heparin-binding motifs is shown in blue. PLAT is the C-terminal β-barrel domain of LPL harboring the lipid binding Trp-rich loop. D: Semi-transparent surface representation of LPL in complex with GPIHBP1. The location of the active site and the PSCK3 cleavage site in LPL is shown. The shared binding site for ANGPTL4 and APOC2 is shown in magenta and the regions exhibiting distinct allosteric changes are shown in orange.

## Material and Methods

### Reagents

Bovine LPL (bLPL) was purified from fresh bovine milk (36) and kindly provided by Dr Gunilla Olivecrona (Umeå University). Human LPL (hLPL) and mouse LPL (mLPL) were produced by transfected *Drosophila* S2-cells and purified as described (37). The coiled-coil domain of ANGTPL4 (residues 1–159 with an N-terminal methionine and a C-terminal 6× His-tag)^1^ was produced in *E. coli* (38). A truncated variant of human PSCK3 produced by *Sf9* cells (>2,000 units/ml) was purchased from Sigma-Aldrich (cat F2677). Mass spectrometry grade porcine trypsin was from Promega. Synthetic peptides representing the N-terminal acidic tail of GPIHBP1, and various truncations thereof were obtained at a purity of >95% from TAG-Copenhagen A/S and they are listed in supplemental Table S1. A GPIHBP1^1–33^ peptide carrying a tyrosyl-sulfation (Tyr^18^) was prepared as described (28). Due to the lack (or paucity) of aromatic amino acids, peptide concentrations were determined by absorbance at 214 nm using calculated molar extinction coefficients (39). Defined DP4 heparin fragments with a terminal biotin moiety and with zero or eight SO_4_ groups were purchased from Iduron and their structures are shown in supplemental Table S1.

### Purified recombinant GPIHBP1

Recombinant and secreted versions of human GPIHBP1^1–131/R38G^ were produced in *Drosophila* S2-cells as fusion proteins with an N-terminal uPAR domain III that served as a purification tag (40). After affinity purification, the tag was removed by enterokinase cleavage, and GPIHBP1^1–131/R38G^ was purified to homogeneity by cation-exchange chromatography as described (18). For practical reasons, all recombinant GPIHBP1 proteins with a uPAR D3-tag contained an Arg^38^ to Gly substitution; that amino acid substitution provided a higher efficiency of correct tag removal by enterokinase without altering LPL-binding properties (18). For simplicity, the Arg^38^ to Gly mutation will not be specified in the following sections, as it was present in all GPIHBP1 variants—except for the wild-type GPIHBP1^1–131^ protein that was used for EDC crosslinking. This protein was expressed without the uPAR D3-tag and purified by affinity chromatography using an immobilized rat anti-human GPIHBP1 monoclonal antibody RE3 (41). A truncated version of GPIHBP1 (GPIHBP1^34–131^) lacking the acidic tail resulted from a second unintentional cleavage after Arg^33^ and was obtained as a useful byproduct from the cation-exchange chromatography (18). Two new D3-tagged GPIHBP1 constructs were obtained from VectorBuilder Inc. and the corresponding proteins were expressed by *Drosophila* S2-cells and purified by existing protocols (18). These two GPIHBP1 mutants had distinct charge neutralizations with all acidic residues (Glu/Asp) being replaced by the corresponding amines (Gln/Asn) at either residues 1–17 (GPIHBP1^*Cluster1-neutral*^) or residues 19–30 (GPIHBP1^*Cluster2-neutral*^), as illustrated in Fig. 1A.

### Protein stability by differential scanning fluorimetry

The thermostability of bLPL in the presence or absence of GPIHBP1 was measured by differential scanning fluorometry (nano-DSF) with a Prometheus Panta (Nanotemper Technologies) using a previously published method (22). In brief; 10 μM bLPL alone or in the presence of 15 μM GPIHBP1^1–131^, 15 μM GPIHBP1^*Cluster1-neutral*^, or 15 μM GPIHBP1^*Cluster2-neutra1*^ were prepared in 10 mM HEPES, 150 mM NaCl (pH 7.4) and heated in capillaries with a ramping temperature of 1°C/min from 15−95°C. Turbidity was measured by assessing back-reflection and was used as a surrogate marker for thermal unfolding (20). All samples were measured in triplicates.

### PCSK3 cleavage of LPL

To determine if charge-variants of GPIHBP1 confer distinct resistance to ANGPTL4-facilitated cleavage of LPL after Arg^324^ by PCSK3 (33), we incubated purified bLPL (20 μM) with PCSK3 (4 units) for 0–240 min, either alone or in the presence of ANGPTL4 (2 μM) and/or GPIHBP1 (25 μM) at 25°C in 10 mM 2-(N-morpholino) ethane sulfonic acid (MES), 150 mM NaCl, 1 mM CaCl_2_, pH 7.0. We tested four different GPIHBP1 variants: GPIHBP1^1–131^, GPIHBP1^*Cluster1-neutral*^, GPIHBP1^*Cluster2-neutral*^, and GPIHBP1^34–131^ (LU domain only). PCSK3 cleavage was stopped by boiling in SDS-PAGE sample buffer (reduced and alkylated) and the degree of cleavage was assessed by SDS-PAGE using 12% Bis-Tris SDS-PAGE gels followed by densitometric scanning of the corresponding Coomassie blue–stained SDS-polyacrylamide gels.

### Lipase activity assay

To determine lipase activity, 90 μl incubation mixture containing 166 mM NaCl, 28 U/ml heparin, 10% (w/v) fatty acid-free serum albumin, 250 mM Tris-HCl (pH 8.5), 0.16% intralipid, and 6% (v/v) chicken serum was mixed with 60 μl of sample buffer consisting of 150 mM NaCl, 0.1% (w/v) fatty acid-free serum albumin, 0.01% (v/v) Triton X-100, 10 mM HEPES (pH 7.4) and 15 nM bLPL. After 25 min incubation, non-esterified fatty acids (NEFA) released by LPL were quantified using the NEFA-HR kit according to manufacturer protocol (Wako Chemicals).

### LPL unfolding as assessed by hydrogen-deuterium exchange mass spectrometry

To measure the impact of synthetic GPIHBP1 tail peptides on LPL-unfolding, we incubated 10 μM bLPL with or without 5 μM synthetic peptides for 45 min at 25°C in 10 mM Na_2_HPO_4_, 150 mM NaCl (pH 7.4) followed by a 10 s pulse-labeling in deuterium oxide (10-fold dilution in the same buffer containing 70% D_2_O). Further deuterium exchange was quenched by mixing with 1 volume ice-cold quenching buffer (100 mM Na_2_HPO_4_, 0.8 M TCEP, 2 M urea in H_2_O, pH 2.5). Samples were subjected to online pepsin digestion and the generated peptides were separated by a SynaptG2 electrospray ionization mass spectrometer (Waters) following a published procedure (18). EX1 exchange kinetics with the emergence of bimodal isotope envelopes for peptides representing the catalytic triad (e.g., residues 131–165) was used as a proxy for LPL unfolding (18, 19, 21, 22, 28, 42). The fraction of folded LPL was calculated by binomial fitting using HX-Express2 (20) to the bimodal peak of the peptide encompassing residues 131–165. The fraction of the low mass population of this peptide corresponds to the fraction of folded LPL.

### GPIHBP1-mediated extraction of LPL from HSPGs

To mimic electrostatic conditions found in the sub-endothelial spaces where surface-bound heparan-sulfate proteoglycans (HSPGs) are abundant, we designed a surrogate sensor surface using a streptavidin-coated CM5 sensor chip to capture high densities of biotinylated synthetic heparin dp4 fragments (28, 29). The reference surface was loaded with a neutral and non-sulfated fragment (M09 S00 from Iduron), while the active surface was loaded with a negatively charged fragment (M09 S08a from Iduron) containing *N*- and *O*-6-linked sulfations on each of the four glucosamine residues in the DP4 fragment. Only the highly sulfated oligosaccharide bound LPL, and those interactions were transient with high off-rates. Despite the very transient nature of this binding, we created a stable reservoir of hLPL on the sensor surface by combining the fast kinetics of polyelectrolyte interactions with severe mass transport limitation—the latter phenomenon impedes LPL escape from the surface due to the fast rebinding that is favored by the high densities of immobilized heparin fragments (300 fmol/mm^2^). For each experiment, 12.5 nM hLPL was loaded for 100 s at a flow rate of 20 μl/min in 10 mM Hepes (pH 7.4), 150 mM NaCl, 4 mM CaCl_2_, 10% (vol/vol) glycerol, 0.05% (vol/vol) surfactant P20, 1 mg/ml defatted BSA, 1 μM GPIHBP1^1–33^, and 0.05% (wt/vol) NaN_3_. The low levels of GPIHBP1^1–33^ in the buffer did not interfere with the capture of hLPL to the high surface density of heparin fragments (M09 S08a) and were included to stabilize LPL and promote uniform LPL binding to the sensor chip. This procedure resulted in capture levels ranging from 12–14 fmols LPL/mm^2^.

To compare the ability of GPIHBP1^1–131^ and its charge variants to bind and extract hLPL from this dynamic reservoir, we injected twofold serial dilutions of 1.25–200 nM GPIHBP1^1–131^; 1.25–200 nM GPIHBP1^*Cluster1-neutral*^; GPIHBP1^*Cluster2-neutral*^; 1.25–200 nM GPIHBP1^34–131^; 0.1–1.6 μM GPIHBP1^1–33^ with Tyr^18^–SO_4_; or 1.25–200 nM GPIHBP1^34–131^ in the presence of 1 μM sulfated GPIHBP1^1–33^ for 250 s at 20 μl/min in 10 mM Hepes (pH 7.4), 150 mM NaCl, 4 mM CaCl_2_, 10% (vol/vol) glycerol, 0.05% (vol/vol) surfactant P20, 0.2 mg/ml fatty acid-free BSA, and 0.05% (wt/vol) NaN_3_. At the end of each cycle, two consecutive injections of 10 μl 3 M guanidinium chloride regenerated the chip.

### Zero-length crosslinking of LPL•GPIHBP1 complexes with EDC

To provide an experimental mapping of the interaction landscape for the acidic tail of GPIHBP1 on hLPL, we performed zero-length crosslinking experiments with 1-ethyl-3-(3-dimethylaminopropyl)carbodiimide (EDC). As a first step, we activated carboxylates on GPIHBP by incubating 37 μM of GPIHBP1^1–131^, GPIHBP1^*Cluster1-neutral*^, or GPIHBP1^*Cluster2-neutral*^ with 1.7 mM EDC for 30 s at 22°C in 10 mM MES, 150 mM NaCl, pH 5.0. The cross-linking process was then initiated by adding purified hLPL in 10 mM MES, 150 mM NaCl, pH 5.0 to a final concentration of 21 μM GPIHBP1, 4 μM hLPL, and 1 mM EDC. Based on crosslinking efficiencies in pilot experiments, the final reactions proceeded for 2 h for GPIHBP1^1–131^ and 4 h for both GPIHBP1^*Cluster1-neutral*^ and GPIHBP1^*Cluster2-neutral*^.

### Identification of EDC crosslinking sites between GPIHBP1 and LPL

To identify EDC crosslinking sites between GPIHBP1 and hLPL, we excised Coomassie-stained bands representing crosslinked products from the polyacrylamide gel. The gel pieces were de-stained, trypsin digested, and the generated peptides were extracted in low-binding tubes (43). In brief, gel pieces were destained by incubation for 30 min in a thermomixer at 300 rpm in 100 μl of 100 mM ammonium bicarbonate with 50% (v/v) acetonitrile. Gel pieces were shrunken by incubation in 500 μl acetonitrile and were subsequently submerged in 10 mM ammonium bicarbonate with 10% (v/v) acetonitrile containing 13 ng/μl trypsin and incubated on ice for 30 min (for the O18-labeling, normal water was substituted with H_2_^18^O). Overnight digestion was carried out in a thermomixer, 400 rpm at 28°C, pH 8. To minimize trypsin-catalyzed back-exchange of O18-labeled C-termini during peptide extraction, trypsin was inactivated by a 10 min incubation at 80°C before tryptic peptides were extracted by incubating the gel pieces in 100 μl extraction buffer (5% formic acid/acetonitrile; 1:2 (v/v)) at 37°C for 15 min on thermomixer 400 rpm (44). Extracted peptides were dried using vacuum centrifugation.

After reconstitution in 60 uL 0.1% formic acid, the extracted peptides were desalted using an Oligo R2/R3 reversed-phase purification, as described previously (45). Bound peptides were eluted with a gradient of 30%, 50%, and 70% acetonitrile in 0.1% formic acid, dried by vacuum centrifugation and stored at −20°C until LC-MS/MS analysis.

LC-MS/MS analysis was carried out using an Easy-LC nano-HPLC system (Proxeon) connected to an Orbitrap Exploris 480 mass spectrometer (Thermo Fisher Scientific). Samples were loaded onto a trap column and then separated using two reversed-phase analytical columns: a 5 cm × 75 μm Reprosil-Pur C18-AQ column (5 μm particle size) and an 18 cm × 75 μm Reprosil-Pur C18-AQ column (3 μm particle size), both from Dr Maisch GmbH. The mobile phases for gradient elution were solvent A (0.1% formic acid in water) and solvent B (90% acetonitrile with 0.1% formic acid) and a 120 min gradient from 0% to 40% solvent B eluted the peptides at a flow rate of 300 nl/min. Mass spectrometry was performed in positive ion mode, with nano-electrospray ionization (ESI) applied at a spray voltage of 2300 V for positive ions. The ion transfer tube temperature was maintained at 290°C. Data acquisition was conducted in data-dependent acquisition (DDA) mode, with MS scans covering an m/z range of 350–2000 at a resolution of 120,000 using the Orbitrap analyzer. The automatic gain control (AGC) target for full MS scans was set at 3 × 10^6^ with a maximum injection time of 50 ms. For MS/MS analysis, the 15 most intense ions were selected for higher-energy collision dissociation (HCD) with an isolation window of 1.0 Da. The normalized collision energy (NCE) was set at 35%, and fragment ions detection in the Orbitrap at a resolution of 30,000. The AGC target for MS/MS was 3 × 10^5^, with a maximum injection time of 150 ms. Dynamic exclusion was activated for 20 s to avoid repeated analysis of the same precursor ions, with a mass tolerance of ±10 ppm. The instrument was operated in cycle time mode, and data was recorded in profile or centroid mode.

Raw data for crosslinked peptide identifications were processed and analyzed using Mascot Distiller version 2.8.2 (Matrix Science Ltd) and the Mascot server version 3.0 as described (46). First, we configured an EDC crosslink-specific method (delta mass of the intact crosslink: −18 Da) for human LPL and GPIHBP1 with accession numbers (P06858, Q8IV16) in the Mascot following Matrix Science’s guidelines. Fragment ions were de-charged by Mascot Distiller 2.8.2. With the Mascot configuration editor, we created databases for hLPL and GPIHBP1 omitting signaling peptides but including the R324A mutation in hLPL and the relevant charge neutralizations in the GPIHBP1 variants. Files generated by Distiller were searched against these databases and contaminant FASTA files using Mascot Server 3.0. Databases were searched for inter- and intra-crosslinks using the following parameters: precursor mass tolerance of 5 ppm, product ion mass tolerance of 5 ppm, fixed modification of carboxyamidomethylation on cysteine, and allowance for 2 missed cleavages for Trypsin. Variable modifications included methionine oxidation, pyroglutamate formation at glutamic acid (Q), sulfation at tyrosine (Y), and methionine (M) oxidation. For the O18-labeled samples, incorporation of O18 labels at any C-termini was included as an additional variable modification.

A significance threshold of *P* < 0.001 was applied with at least one significant unique sequence and a Mascot ion score greater than 80 and selected crosslinks were validated manually including the incorporation of at least two ^18^O-labels in their C-termini. The results were exported in xiVIEW CSV format, with peak lists in MGF and sequences in FASTA, for viewing in XiView. Filtered crosslinks were exported in CSV format (47).

### Molecular modeling of GPIHBP1 and LPL•GPIHBP1 complexes

Models for GPIHBP1 variants and their complexes with hLPL protein were generated with AlphaFold 3. The modeling returns a total of five predicted binding poses. Among those, the most confident complex was chosen based on template modeling scores (pTM and ipTM). In some regions, the scores were low due to the intrinsically disordered region in GPIHBP1. The sequences submitted to the AlphaFold 3 server were obtained from Uniprot (accession codes: P06858 for hLPL and Q8IV16 for hGPIHBP1). Sequences were manually modified for charge-neutralized variants, Tyr^18^ phosphorylation was used as a mimic of Tyr^18^ sulfation (28), and N- and C-terminal signal sequences were removed.

## Results

### Impact of negative charges in peptides from GPIHBP1’s disordered tail on LPL stability

**Fig. 2:**
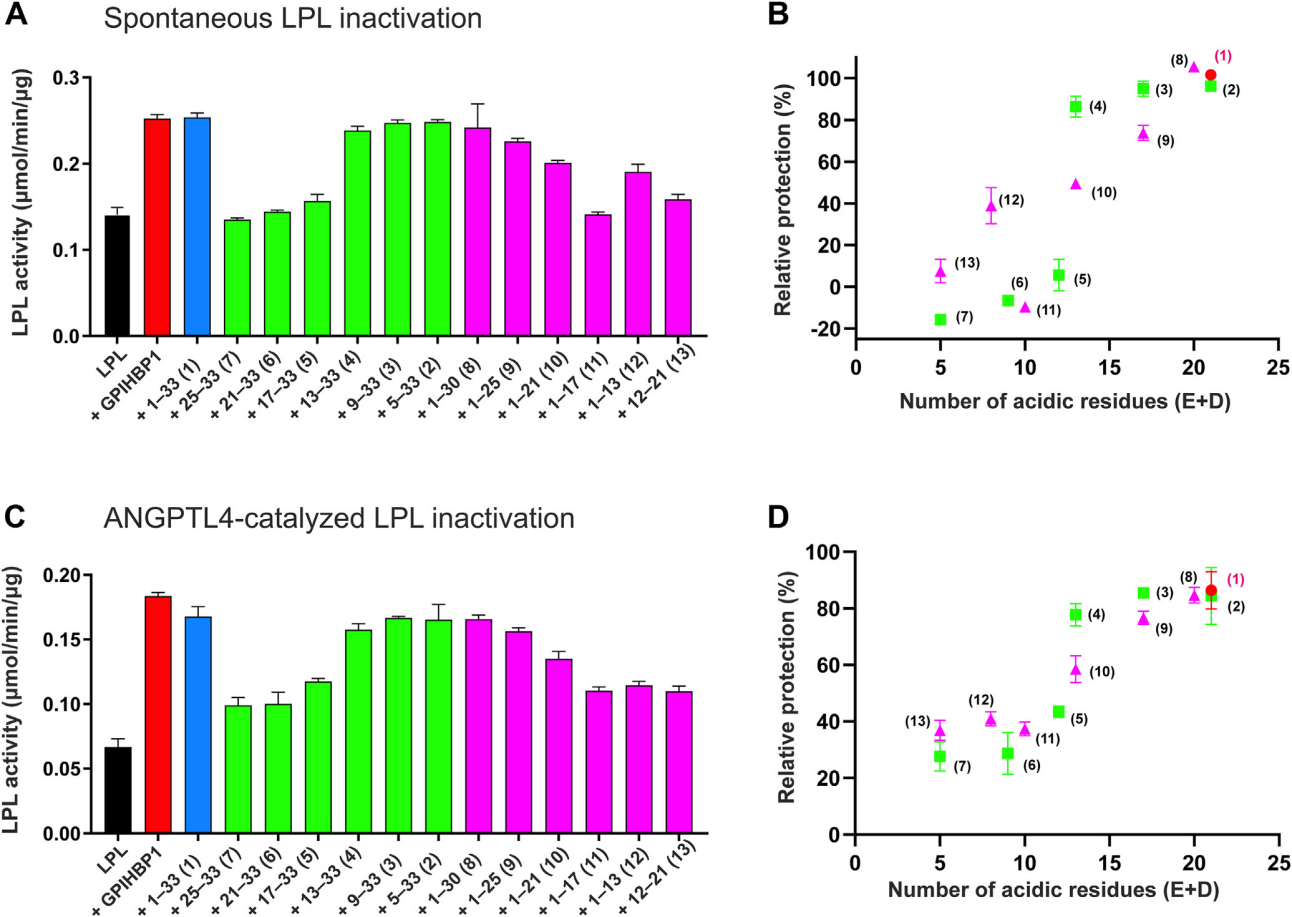
LPL activity after incubation with synthetic peptides covering the acidic tail of GPIHBP^1–33^ Purified bLPL (15 nM) was preincubated either alone or in the presence of 1 μM synthetic peptides or 150 nM GPIHBP1 for 20 min in 10 mM HEPES, 150 mM NaCl pH 7.4 at 22°C before LPL activity was assessed using Intralipid® as substrate. Numbers refer to the GPIHBP1 peptides listed in supplemental Table S1; N-terminal truncations are highlighted in *green* while C-terminal truncations are *pink*. A: Residual LPL activity after preincubation with synthetic peptides. B: Relative protection against spontaneous inactivation by 1 μM synthetic peptides compared to 150 nM GPIHBP1 (100%) and LPL alone (0%) as a function of number of acidic residues in the respective peptides. Experiments delineated in (C and D) are identical to those in (A and B) except that 15 nM bLPL was preincubated with the GPIHBP1 peptides in the presence of 2 nM ANGPTL4 and the synthetic peptides. D: Protection against ANGPTL4-catalyzed LPL inactivation as a function of negative residues in the peptides. The mean ± SD are shown for triplicate measurements.

Earlier studies revealed that the disordered acidic tail of GPIHBP1 protects against spontaneous and ANGPTL4-catalyzed inactivation of LPL (18, 21, 28), but it was not clear from those studies whether this effect was sequence-specific or merely reflected the average electric field strength. To address this question, we preincubated 15 nM purified bLPL for 20 min in the presence of 1 μM of synthetic peptides with different chain lengths and covering distinct regions of the first 33 residues in GPIHBP1 (supplemental Table S1). The residual bLPL activity was subsequently assessed by quantifying the release of non-esterified free fatty acids after a 25-min incubation with a lipid substrate emulsion (Intralipid) using the NEFA2-kit. In this setting, 1 μM GPIHBP1^1–33^ was as efficient as 150 nM GPIHBP1^1–131^ in preserving bLPL activity against spontaneous inactivation (Fig. 2A). Decreasing the number of acidic residues from 21 to only 5 by stepwise truncations of GPIHBP1^1–33^ led to a reduced LPL activity, which was comparable for C- and N-terminal truncations (Fig. 2B). A similar correlation was also noted for the protection against ANGPTL4-catalyzed LPL inactivation by the same ensemble of synthetic acid tail peptides (Fig. 2C, D). Thus, our data favors the importance of the electrical field strength over a sequence-specific charge patterning in preserving LPL activity. Under these conditions, negative charges above −11 of the synthetic GPIHBP1 peptides proved sufficient for optimal protection of LPL activity as revealed by the progressive loss in potency of peptides 4−6 and 9−11 (Fig. 2B, D).

**Fig. 3:**
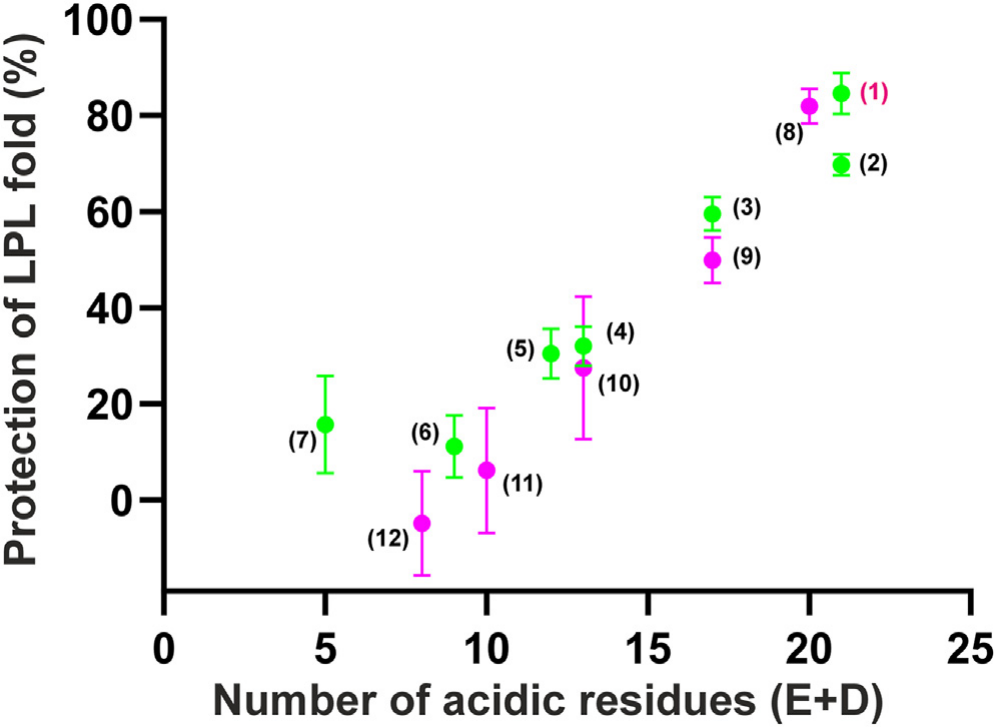
Charge-dependent alleviation of spontaneous LPL-unfolding by peptides from GPIHBP^1–33^. In these studies, we incubated bLPL alone or in the presence of synthetic peptides from GPIHBP1’s acidic tail for 45 min at 25°C in 10 mM PBS (pH 7.4) followed by a 10 s pulse-labelling in 70% D_2_O. Quenched and reduced samples were finally digested by pepsin and analyzed by mass spectrometry. The emergence of bimodal isotope envelope for residues 131–165 (containing 2 residues for LPL’s catalytic triad) is the result of a correlated (EX1) deuterium exchange and is considered a proxy for LPL unfolding (18, 21). Shown is the calculated fraction of folded LPL in the presence of truncated peptides relative to intact GPIHBP^1–131^ (defined as 100% protection) and the absence of added peptides (defined as 0% protection). The mean ± SEM are shown for triplicate measurements. Numbers refer to the peptides listed in supplemental Table S1.

As an orthogonal method, we used pulse-labeling hydrogen-deuterium exchange mass spectrometry (HDX-MS) studies to measure the impact of the same acidic tail peptides on the spontaneous unfolding of LPL. Aligned with earlier studies, we used the emergence of bimodal isotope envelopes in a peptic peptide from LPL’s catalytic pocket (e.g., 131–165) as a proxy for unfolding of the α/β-hydrolase domain (18, 21, 42). Pre-incubation of bLPL for 45 min at 25°C with the free peptides revealed that reducing the number of acidic residues in GPIHBP1^1–33^ by truncation gradually aggravated the spontaneous unfolding of LPL (Fig. 3). This provided a mechanistic explanation for the loss of lipase activity observed in Fig. 2B i.e. caused by an irreversible unfolding of the active site in LPL.

In aggregate, our studies suggested that the average field strength from the synthetic acid tail peptides was more important than the actual sequence patterning for their ability to mitigate LPL inactivation by unfolding. Notwithstanding this conclusion, it is important to emphasize that the conformational landscape of the acidic disordered tail in GPIHBP1^1–131^ is restricted when it is tethered to the LU domain. Complex formation between LPL and the LU-domain of GPIHBP1^1–131^ enforces an additional positional constraint to the disordered acidic tail by placing charge-cluster 2 adjacent to the basic patch on LPL—confinement that is not imposed on the free acidic tail peptides. Subsequent experiments were therefore conducted with purified recombinant GPIHBP1 proteins having mutations that neutralize either of the two acidic regions in the disordered tail (Fig. 1A).

### Expression, purification, and characterization of charge-neutralized GPIHBP1 mutants

To study the impact of charge distributions in the context of intact GPIHBP1, we expressed two GPIHBP1 mutants denoted *Cluster1-neutral* and *Cluster2-neutral* (Fig. 1A) in *Drosophila* S2 cells as fusion proteins with uPAR D3 (40). After purification and removal of the D3 tag (18), we obtained pure GPIHBP1^*Cluster1-neutral*^ and GPIHBP1^*Cluster2-neutral*^ proteins that bound hLPL, bLPL, and mLPL as judged by native PAGE (supplemental Fig. S1). Analyses by electrospray ionization mass spectrometry revealed that all GPIHBP1 proteins contained one bi-antennary N-linked glycosylation. Posttranslational sulfation of Tyr^18^ in GPIHBP1^1–131^ (28) was absent in GPIHBP1^*Cluster2-neutral*^ and present in less than 40% of GPIHBP1^*Cluster1-neutral*^ (supplemental Fig. S2). That finding is well aligned with predictions from Sulfinator (48), which highlight Tyr^18^ as a likely sulfation site in wild-type GPIHBP1^1–131^, but neither GPIHBP1^*Cluster1-neutral*^ nor GPIHBP1^*Cluster2-neutral*^ were predicted by this algorithm to be sulfated at Tyr^18^. Finally, charge neutralizations of the two acidic tail clusters in GPIHBP1 did not cause gross alterations in their overall secondary protein structure as assessed by circular dichroism and molecular modeling with AlphaFold 3 (supplemental Fig. S3).

### LPL inactivation by ANGPTL4 depends on charge patterns in GPIHBP1

**Fig. 4:**
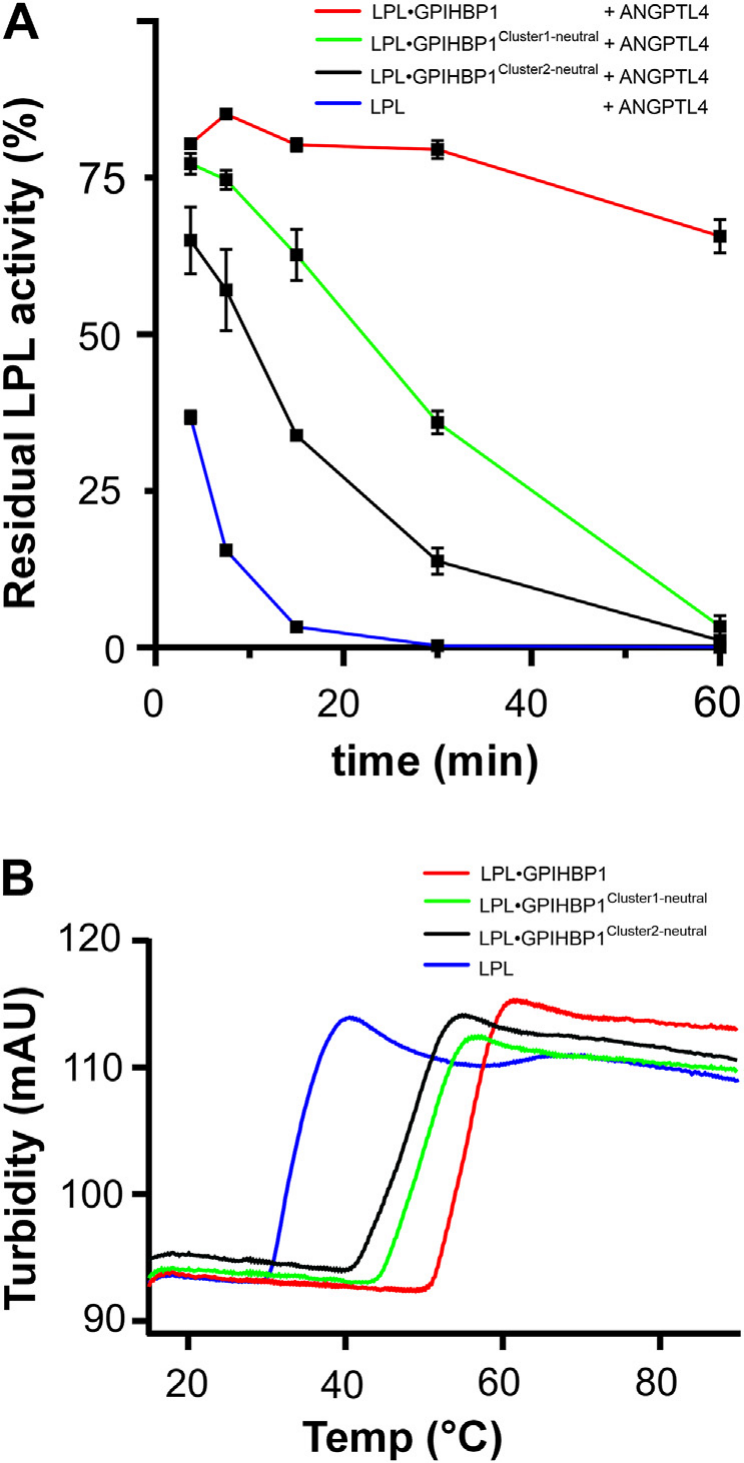
GPIHBP1 clusters 1 and 2 have distinct impacts on LPL stability and inactivation by ANGPTL4. A: Residual LPL activity after pre-incubating 30 nM bLPL and 30 nM ANGPTL4 alone or in the presence of 300 nM GPIHBP1^1–131^, 300 nM GPIHBP1^*Cluster1-neutral*^, or 300 nM GPIHBP1^*Cluster2-neutral*^ at room temperature in 10 mM HEPES, 150 mM NaCl, 0.1 mg/ml fatty acid free BSA, pH 7.4. LPL activity was measured by the release of free fatty acids from Intralipid using the NEFA 2 assay. The residual LPL activity is plotted relative to 30 nM bLPL and 300 nM GPIHBP1^1–131^ with no pre-incubation. Shown are the means ± SD for measurements in triplicate. B: Thermostability of 10 μM bLPL in the presence of 15 μM of the different GPIHBP1 charge variants using *T*_*turbidity*_ measured by Prometheus Panta™ as a surrogate marker for unfolding (20).

Next, we probed if recombinant GPIHBP1^1–131^ with defined neutralizations of charge-clusters 1 or 2 had distinct impacts on their ability to protect bLPL from ANGPTL4-mediated inactivation. To address this question, we pre-incubated 30 nM bLPL, 30 nM ANGPTL4, and 300 nM GPIHBP1 at room temperature for up to 60 min and compared the time-dependent decay of LPL activity in the presence of the different GPIHBP1 variants (Fig. 4A). Despite GPIHBP1^1–131^ instigated a long delay in LPL inactivation by ANGPTL4, the two charge-neutralized variants were less potent. In contrast to the free acidic tail peptides (Fig. 2), the charge distribution in the acidic tail was now important for the protective effect of intact GPIHBP; with GPIHBP1^*Cluster1-neutral*^ having greater efficacy than GPIHBP1^*Cluster2-neutral*^ (Fig. 4A). Thus, charge-cluster 2 had a dominating role in the protection of bLPL against ANGPTL4-mediated inactivation. Differential scanning fluorimetry (DSF) revealed that stabilization of LPL’s α/β-hydrolase domain also correlated to the charge patterning of GPIHBP1’s acidic tail (Fig. 4B). Using turbidity (protein aggregation) as a proxy for LPL stability (20), we found that GPIHBP1 charge-clusters 1 and 2 had distinct capacities to mitigate thermal unfolding and aggregation of bLPL: *T*_*turbidity*_ is 55.8 ± 0.1°C for LPL with GPIHBP1^1–131^, 50.4 ± 0.2°C for LPL with GPIHBP1^*Cluster1-neutral*^, 48.8 ± 0.1°C for LPL with GPIHBP1^*Cluster2-neutral*^, and 32.6 ± 0.1°C for LPL alone. In aggregate, these studies highlighted the importance of negative charges in cluster 2 for preserving LPL stability and activity. Nonetheless, it was also clear that additional contributions from charges located in cluster 1 were needed to attain the full protective capacity of GPIHBP1^1–131^ against LPL unfolding and aggregation (Fig. 4).

### Protection against ANGPTL4-facillitated PSCK3 cleavage of LPL

**Fig. 5:**
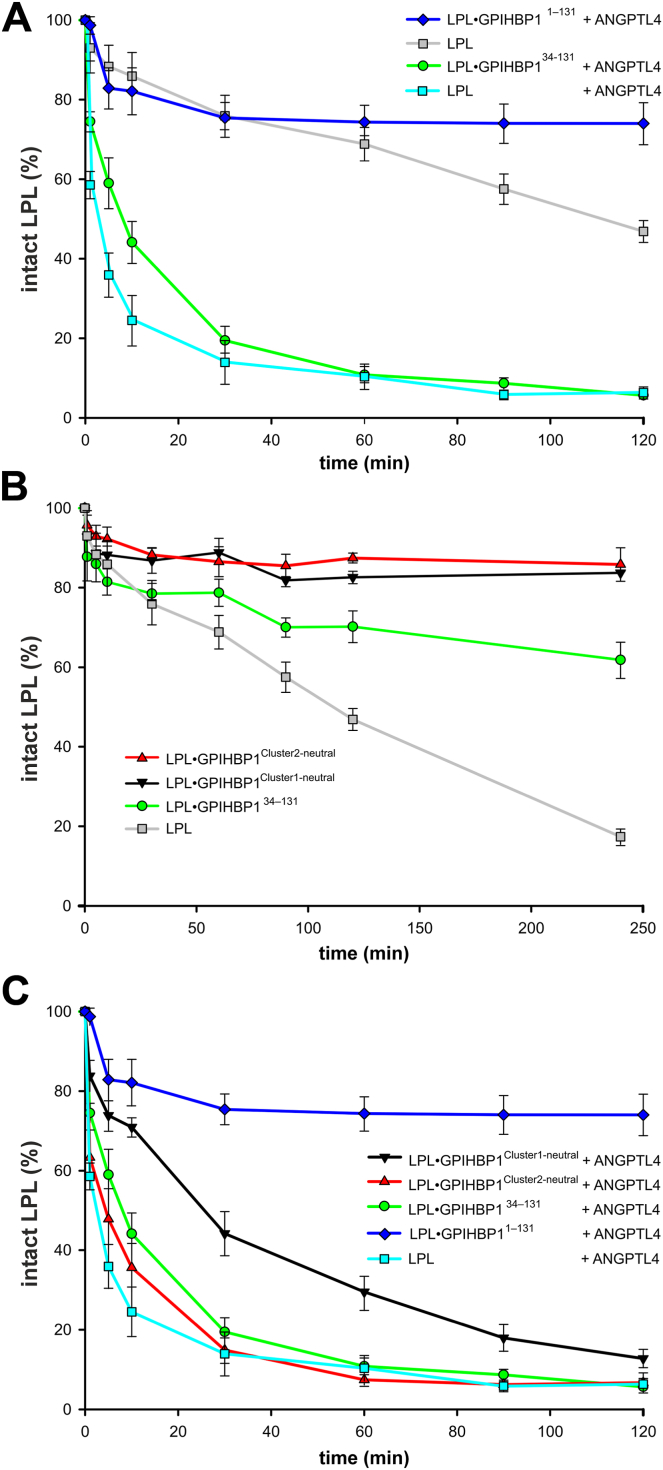
GPIHBP1 reduces ANGPTL4-facillitated PSCK3 cleavage of LPL. 20 μM bLPL was incubated with 4 units of PSCK3 for 0–240 min at 25°C and the extent of LPL cleavage after Arg^324^ was assessed by densitometric scanning of Coomassie–blue stained polyacrylamide after SDS-PAGE of reduced and alkylated samples. A: PSCK3 cleavage of LPL is accelerated by the presence of 2 μM ANGPTL4—present at sub-stoichiometric amounts compared to bLPL (20 μM). 25 μM intact GPIHBP1, but not GPIHBP1^34–131^ mitigates PSCK3 cleavage of LPL. B: Both GPIHBP1^Cluster1-neutral^ and GPIHBP1^Cluster2-neutral^ rescue LPL from cleavage by PSCK3 in the absence of ANGPTL4 and both are more efficient than GPIHBP1^34–131^. C: in the presence of 2 μM ANGPTL4, 25 μM GPIHBP1^Cluster1-neutral^ protects LPL from PSCK3 cleavage more efficiently than the GPIHBP1^*Cluster2-neutral*^, which indicates a more prominent role of acidic cluster 2 in protecting LPL from ANGPTL4-catalyzed unfolding and subsequent PSCK3 cleavage. Shown are the mean values (±SEM) for intact LPL relative to that present at time 0 min for 3–5 independent time-course experiments. Some curves appear in multiple panels to increase clarity.

Secretion of LPL by adipocytes is modulated in an autocrine manner by ANGPTL4. Elegant studies by Dijk *et al.* (34, 35) showed that adipocytes from *A*ngptl*4*^*−/−*^ mice had increased secretion of active LPL compared to adipocytes from wt mice. In the presence of endogenous ANGPTL4, a significant fraction of LPL was cleaved by PCSK3 in the *trans*-Golgi compartment. Subsequent studies with purified proteins showed that ANGPTL4 sensitized bLPL to PCSK3-mediated cleavage by catalyzing the unfolding of its hydrolase domain (33). The presence of GPIHBP1 dramatically delayed PCSK3 cleavage of LPL (33). To assess the role of the acidic tail in GPIHBP1 on this delay, we incubated 20 μM bLPL with 4 units of PCSK3 either alone or in the presence of 2 μM ANGPTL4. We observed that the presence of sub-stoichiometric amounts of ANGPTL4 reduced the half-life of intact bLPL from 110 min to less than 5 min (Fig. 5A). The presence of 25 μM GPIHBP1^1–131^ blocked PCSK3 cleavage of bLPL whereas 25 μM GPIHBP1^34–131^ had little effect (Fig. 5A). Our results emphasized the essential role of GPIHBP1’s acidic tail in protecting LPL against ANGPTL4-induced cleavage by PCSK3. When LPL cleavage by PCSK3 was triggered by spontaneous unfolding (i.e., without ANGPTL4), both GPIHBP1^*Cluster1-neutral*^ and GPIHBP1^*Cluster2-neutral*^ prevented that cleavage completely (Fig. 5B). However, when PCSK3 cleavage was triggered by ANGPTL4-catalyzed LPL unfolding, GPIHBP1^*Cluster2-neutral*^ provided marginal protection—much like GPIHBP1^34–131^—whereas GPIHBP1^*Cluster1-neutral*^ resulted in significant protection, which prolonged the half-life of intact LPL from less than 5 min to 25 min (Fig. 5C). In aggregate, these experiments demonstrated that charge-cluster 2 played a dominant role in protecting LPL from PCSK3-mediated cleavage.

### Dislodgement of LPL from its HSPG-bound state by GPIHBP1

**Fig. 6:**
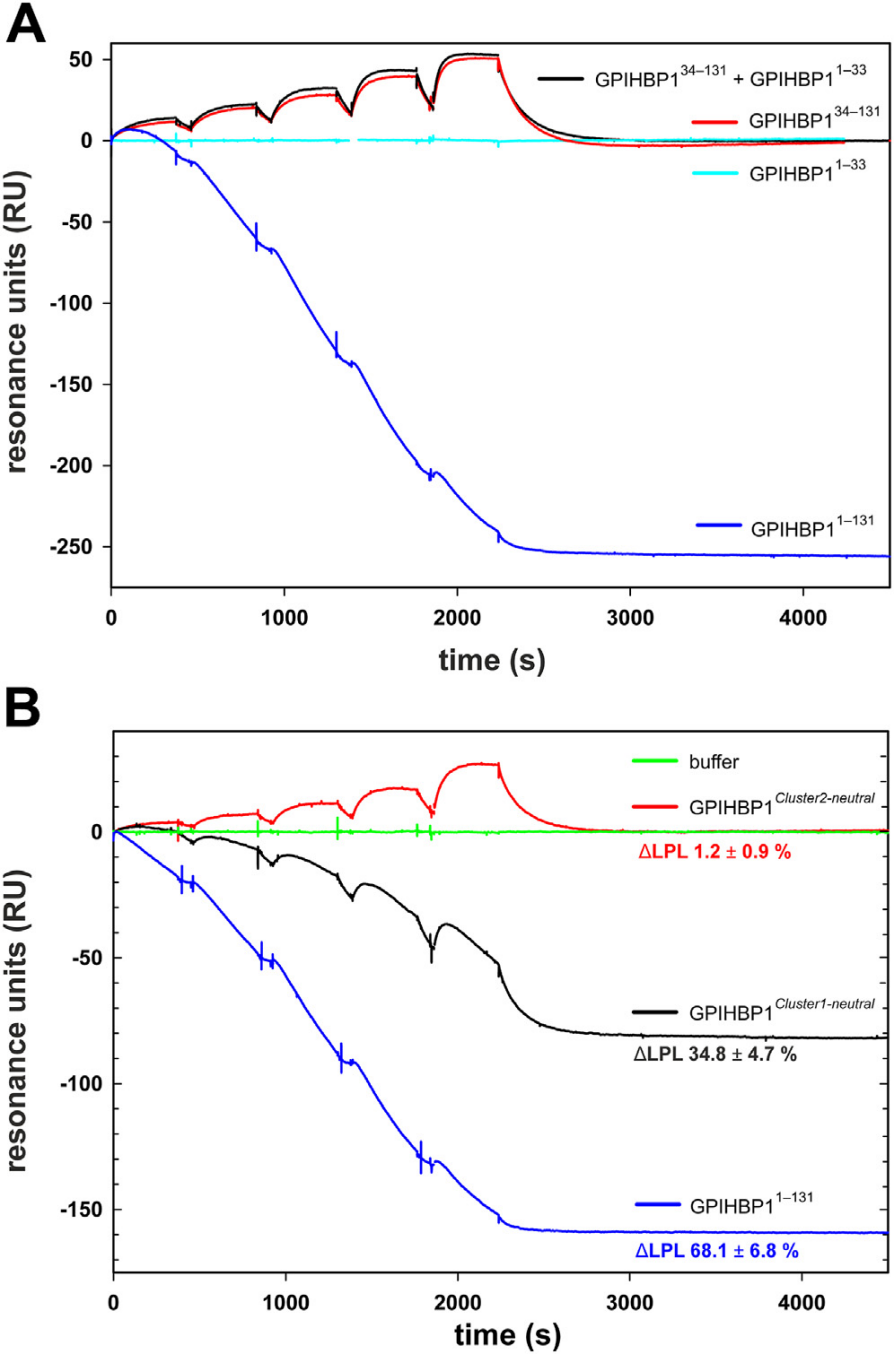
GPIHBP1 acidic cluster 2 is required for extraction of HSPG-bound LPL. A: hLPL binds stably to a high-density surface of immobilized heparan sulfate, and that association was not perturbed by injections of serial twofold dilutions of 0.1–1.6 μM sulfated GPIHBP1^1–33^. Injections of 12.5–200 nM of GPIHBP1^34–131^ whether alone or in the presence of 1 μM sulfated GPIHBP1^1–33^ led to the binding of GPIHBP1^34–131^ to the heparan sulfate-bound LPL, but that interaction did not lead to the competitive displacement of LPL. In contrast, injections of 12.5–200 nM GPIHBP1^1–131^ weakened hLPL’s association to the heparan sulfate surface and led to a pronounced dissociation of LPL. B: Competitive displacement of hLPL tethered to heparan sulfate by serial injections of twofold dilutions of GPIHBP1^1–131^, GPIHBP1^Cluster1-neutral^, or GPIHBP1^Cluster2-neutral^ (12.5–200 nM). Reductions in hLPL levels on the sensor surface after GPIHBP1 injections were: 68.1 ± 6.8% with GPIHBP1^1–131^, 34.8 ± 4.7% with GPIHBP1^Cluster1-neutral^, and 1.2 ± 0.9% with GPIHBP1^Cluster2-neutral^ (mean ± SD, n = 3).

GPIHBP1’s ability to displace LPL from its HSPG-bound state in the sub-endothelial spaces is essential for the *trans*-endothelial trafficking of LPL to the luminal surface of capillary cells (29). The importance of the acidic tail in this process is supported by solid evidence from surrogate in vitro models and in vivo studies in gene-edited mice (28, 29). It was therefore important to determine the relative impact of the two charge-clusters in GPIHBP1 on their ability to disrupt LPL•HSPG interactions. To gain this insight, we measured the ability of different GPIHBP1 charge variants to displace hLPL from a SPR sensor chip surface coupled with a high density of immobilized small heparin-like fragments (28). As shown in Fig. 6A, intact GPIHBP1^1–131^ efficiently dislodged LPL from its electrostatic tether to immobilized HSPG, whereas neither GPIHBP1^34–131^ nor GPIHBP1^1–33^ alone, or in combination, induced any detachment of LPL. Thus, in the absence of a covalent acidic tail extension, GPIHBP1 was unable to detach LPL from HSPGs. Truncated GPIHBP1^34–131^ merely bound the HSPG-bound LPL via its folded LU domain, but that binding did not lead to the competitive displacement of LPL. When GPIHBP1^*Cluster2-neutral*^ was tested, it recapitulated the effect observed for GPIHBP1^34–131^—both variants bound the HSPG-bound LPL with comparable kinetics, but neither dislodged any LPL from the surface. In contrast, GPIHBP1^*Cluster1-neutral*^ displaced as much as 35% of the total amount of surface-bound LPL compared to 68% displaced by intact GPIHBP1^1–131^ (Fig. 6B). In conclusion, charge-cluster 2 is indispensable for GPIHBP1-mediated displacement of LPL from HSPGs. Charge-cluster 1 augments the potency of charge-cluster 2 in this process.

### Mapping the dynamic landscape of the GPIHBP1•LPL interface with zero-length crosslinking

**Fig. 7:**
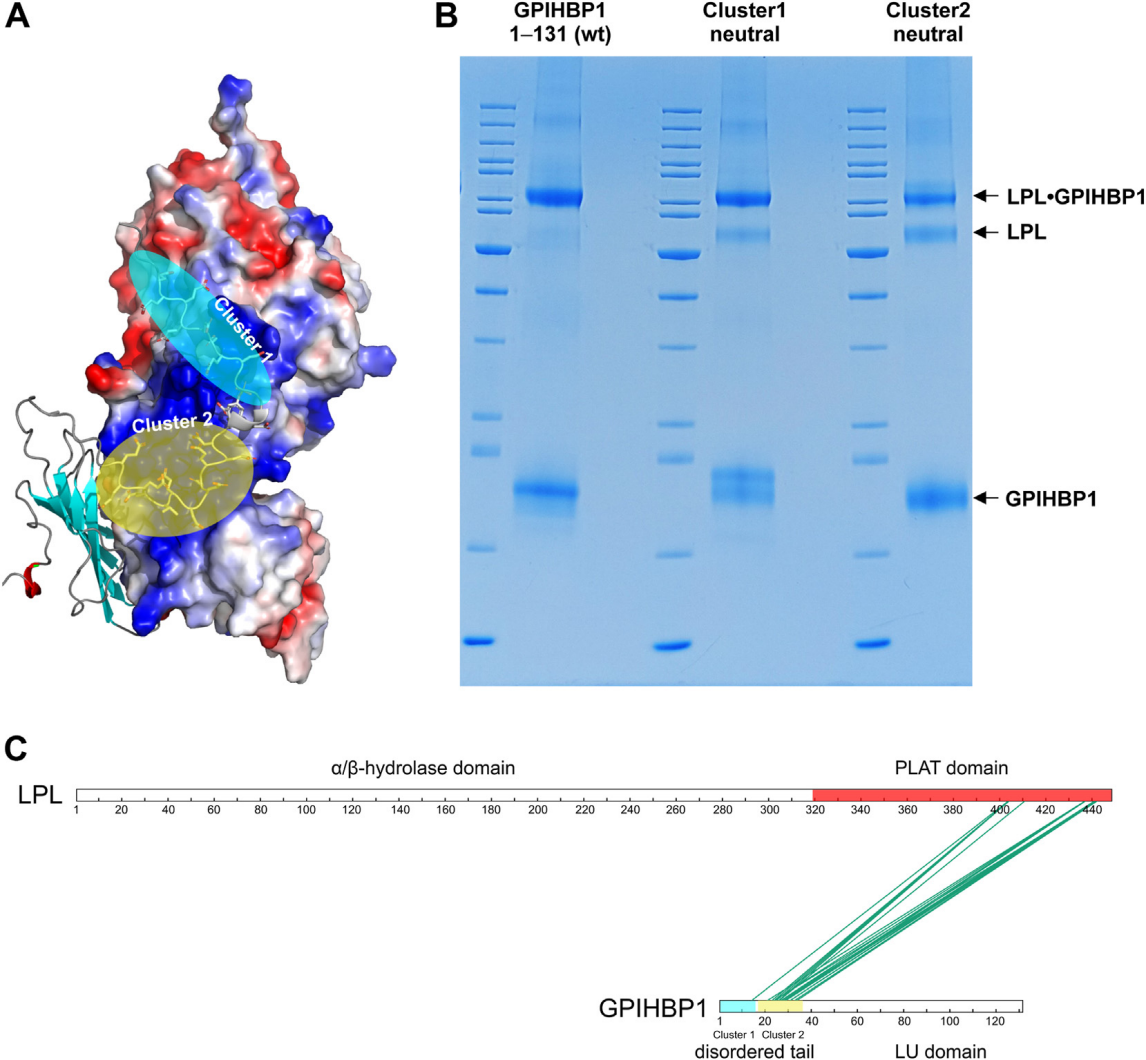
Zero-length crosslinking between LPL and GPIHBP1. A: AlphaFold 3 model of hLPL in complex with GPIHBP1. LPL is shown in a surface representation and colored by electrostatic potential, whereas GPIHBP1 is represented in a cartoon representation with the acidic residues in the disordered tail shown as sticks. Charge cluster 1 is highlighted by the transparent cyan oval whereas charge cluster 2 is highlighted with the transparent yellow oval. B: SDS-PAGE of reduced and alkylated hLPL crosslinked to GPIHBP1^1–131^, GPIHBP1^Cluster1-neutral^, or GPIHBP1^Cluster2-neutral^ using EDC. Protein bands representing LPL•GPIHBP1 complexes were excised and cleaved with trypsin before crosslinks were identified by mass spectrometry. C: xiVIEW representation of the 20 determined EDC crosslinks between GPIHBP1 and hLPL with a high Mascot threshold of ≥80. The individual domains in LPL and GPIHBP1 are highlighted.

To gain structural insights into why charge cluster 2 is a major driving force in the displacement of LPL from HSPGs, we mapped the charge-dependent landscape between hLPL and the acidic tail by AlphaFold 3 modeling and zero-length EDC-crosslinking (Fig. 7). In the model of the LPL•GPIHBP1^1–131^ complex (Fig. 7A), charge-cluster 2 formed extensive ionic contacts with densely packed basic residues in the upper region of LPL’s C-terminal β-barrel including the interface with the α/β-hydrolase domain *i.e.*, Arg^279^, Arg^282^, Lys^319^, Arg^405^, Lys^407^, Lys^414^, Lys^441^, Lys^445^. In contrast, charge-cluster 1 formed a lesser dense interaction with a basic patch on the α/β-hydrolase domain *i.e.*, Arg^263^, Lys^280^, Arg^282^, Lys^296^, and Arg^297^. To validate this model experimentally, we used EDC-based zero-length crosslinking introducing covalent amide bonds between primary amines and carboxylates in hLPL and GPIHBP1. Crosslinking efficiency was high and correlated roughly with the number of ionic contacts depicted by AlphaFold 3 *i.e.*, GPIHBP1^1–131^ > GPIHBP1^*Cluster1-neutral*^ > GPIHBP1^*Cluster2-neutral*^ (Fig. 7B). Coomassie-stained bands corresponding to crosslinked adducts of hLPL and GPIHBP1 were excised from SDS-PAGE gels, destained, and cleaved with trypsin in the presence or absence of ^18^O-labelled water. Crosslinked peptides were identified by Mascot searches and subsequently validated by the increased incorporation of ^18^O-label into their carboxyl-termini by trypsin digestion in ^18^O-labeled water (supplemental Fig. S4). Using a high cut-off Mascot ion score of 80, we identified 16–22 unique crosslinks between hLPL and the acidic tail of GPIHBP1^1–131^ (Fig. 7C and supplemental Table S2), GPIHBP1^*Cluster1-neutral*^ (supplemental Fig. S6), and GPIHBP1^*Cluster2-neutral*^ (supplemental Fig. S7). While LPL-crosslinks with GPIHBP1^*Cluster1-neutral*^ largely recapitulated those found with wildtype GPIHBP1^1–131^, LPL-crosslinking sites with GPIHBP1^*Cluster2-neutral*^ were more diffusely distributed indicating that this charge variant had a less focused binding interface with hLPL (supplemental Figs. S6, S7, and supplemental Table S2).

## Discussion

Numerous genetic and biochemical studies have highlighted the essential role of GPIHBP1 in intravascular lipolysis (19, 30, 49). If the function of GPIHBP1 is disabled by either bi-allelic loss-of-function variants (50, 51) or by inhibitory autoantibodies (8) the affected individuals develop severe hypertriglyceridemia with a risk for life-threatening pancreatitis. A central and indispensable role of GPIHBP1 in this process is to facilitate *trans*-endothelial transport of LPL from its HSPG-bound state in the interstitial spaces to its site of action in the capillary lumen (25). At the luminal endothelial surface, LPL drives margination of TRLs followed by subsequent hydrolysis of their triglyceride cargo—irrespective of whether LPL remained bound to GPIHBP1 or was captured by the lining glycocalyx after dissociation from GPIHBP1 (52, 53). *Trans*-endothelial trafficking of LPL is thus key to intravascular lipolysis, and it exploits the unique protein topology of GPIHBP1. This small glycolipid-anchored membrane protein comprises a C-terminal folded LU domain (26) and a disordered, highly acidic N-terminal tail (18). That protein topology renders GPIHBP1 a “functional dipole”. The folded LU-domain provides a stable binding interface with LPL’s C-terminal β-barrel domain (Fig. 1), which defines the half-life of the LPL•GPIHBP1 complex with a *k*_*off*_ of 7.3 × 10^−3^ s^−1^ (27, 28). In contrast, the disordered acidic tail forms highly dynamic charge-charge interactions with LPL’s heparin-binding motifs, which greatly increases the association rates with a *k*_*on*_ of 3 × 10^8^ M^−1^s^−1^ (28). Polyelectrolyte interactions with GPIHBP1’s acidic tail also protect LPL activity by limiting spontaneous protein unfolding (18), ANGPTL-catalysed unfolding (21, 22, 42), and PSCK3-mediated cleavage (33, 35, 54). The composite nature of the LPL•GPIHBP1 binding interface is particularly important for *trans*-endothelial LPL trafficking. In this pathway, the acidic disordered tail of GPIHBP1 is required to outcompete the electrostatic tethering of LPL to HSPGs in the sub-endothelial spaces by competitive displacement. This process is most likely initiated by GPIHBP1’s acidic tail invading the binding interface of LPL•HSPG complexes thus resulting in the formation of a transient ternary complex (as observed in Fig. 6). Reinforced by the relatively long-lived interaction between LPL and GPIHBP1’s folded LU-domain, this binding mechanism enables the efficient dislodgment of LPL from its HSPG-tether (28, 29). The mechanism outlined in the current study, where GPIHBP1 drives a competitive displacement of LPL from HSPGs, resembles the polyelectrolyte competition that was recently proposed to drive prothymosin-mediated displacement of histone H1 from the nucleosome (55). In that study, competitive displacement by highly charged and intrinsically disordered proteins was proposed to be a widespread mechanism to regulate *nuclear* interactions. Our current study demonstrates, however, that this mechanism is not merely confined to the nuclear and cytoplasmic compartments, but also operates in the extracellular environment where it controls intravascular lipolysis by enabling *trans*-endothelial trafficking of LPL (28, 29). In this setting, competitive displacement by GPIHBP1 even evolved to act in synergy with traditional binding events between folded protein domains thereby enhancing the potency of the polyelectrolyte invasion and subsequent displacement.

In the current work, we also explored the functional importance of the number and distribution of negative charges in GPIHBP1’s acidic disordered tail. With synthetic peptides, we observed that consecutive truncations led to a progressive loss in their ability to mitigate spontaneous and ANGPTL4-catalyzed inactivation of LPL. That decline in potency correlated with the reduction in negative charges (Figs. 2 and 3). There was no clear difference between N- and C-terminal truncations indicating that the mean electric field strength played a more important role than the actual charge distributions in the synthetic peptides. From the protection of ANGPTL4-catalyzed LPL inactivation (Fig. 3D), it appeared that a minimum net negative charge of −11 to −12 was required for optimal protection of LPL activity. This charge requirement aligns well with the evolution of mammalian GPIHBP1s where the acidic tails are 31–45 residues long, contain 14–23 acidic residues, and have an average net negative charge of −0.41 per residue.

Examining the charge patterning of the acidic tail in GPIHBP1 revealed that it harbors two distinct acidic regions (cluster 1 and cluster 2) flanking a sulfated tyrosine (Tyr^18^ in human GPIHBP1). From the sequence logo in Fig. 1B, it is evident that charge-cluster 2 is more conserved than charge-cluster 1. To explore the functional significance of these two acidic clusters, we produced recombinant GPIHBP1^1–131^ variants where charge-clusters 1 and 2 were individually neutralized by replacing their carboxylic acids with the corresponding amides, which reduced net negative charges per residue of the disordered tail from −0.50 in GPIHBP1^1–131^ to −0.26 in GPIHBP1^*Cluster1-neutral*^ and −0.24 in GPIHBP1^*Cluster2-neutral*^.

We found that charge-cluster 2 had a pronounced effect on GPIHBP1’s ability to modulate an array of LPL functions, whereas charge-cluster 1 played an auxiliary role. These effects included mitigating ANGPTL4-catalyzed loss of LPL activity (Fig. 4), delaying PSCK3-mediated LPL cleavage in the presence of ANGPTL4 (Fig. 5), and dislodging LPL from its HSPG-bound state (Fig. 6). The reduced capacity to preserve LPL activity and prevent ANGPTL4-facilitated PSCK3 cleavage is conceivably a direct consequence of GPIHBP1^*Cluster2-neutral*^ being less efficient in stabilizing the native conformation of LPL’s α/β-hydrolase domain. In contrast, the molecular mechanism(s) underpinning the impaired capacity of charge-neutralized GPIHBP1 variants to dislodge LPL from its HSPG-bound state is not merely rooted in the stabilization of the native conformation of LPL. That deficiency likely pertains to a reduced capacity to invade the polyelectrolyte interactions between HSPGs and LPL and initiate LPL dislodgment by competitive displacement. Supporting that idea, profiles of GPIHBP1 binding to and dislodgement of HSPG-bound LPL differed among the tested GPIHBP1 variants (Fig. 6B). While the ternary GPIHBP1•LPL•HSPG complexes were very short-lived in the case of GPIHBP1^1–131^ due to a rapid competitive displacement of LPL•HSPG interactions, GPIHBP1^*Cluster1-neutral*^ formed more long-lived ternary complexes with a slower LPL displacement kinetics inferring that its ability to invade the LPL•HSPG binding interface was diminished. In contrast, stable ternary complexes were formed between GPIHBP1^*Cluster2-neutral*^ and LPL•HSPG complexes and this binding did not lead to any displacement of LPL. This implies that acidic residues at position 19–30 in GPIHBP1’s disordered tail are indispensable for competitive displacement via polyelectrolyte invasion. However, we could not formally exclude an additional effect from a variable level of Tyr^18^ sulfation in our recombinant GPIHBP1 variants (supplemental Fig. S2). Despite this limitation of our studies, we do nevertheless not expect that tyrosine sulfation will have a major impact on GPIHBP1’s ability to dislodge HSPG-bound LPL. Thus, impeding tyrosyl-sulfation in GPIHBP1^1–131^ by a Tyr^18^→ Phe mutation had only a minor negative effect on competitive LPL displacement (28).

**Fig. 8:**
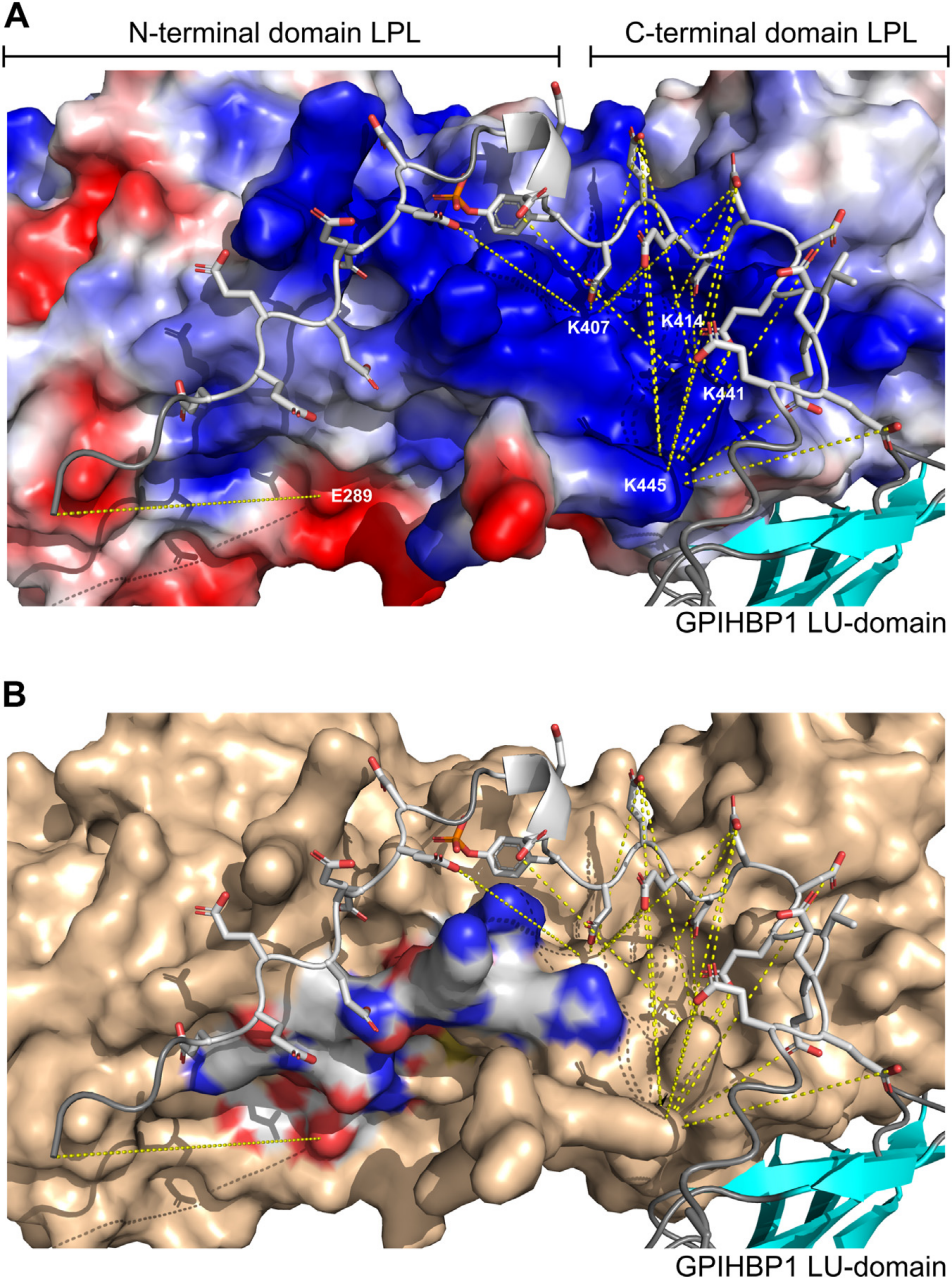
Mapping EDC crosslinks on a model of the binding interface between GPIHBP1’s acidic tail and LPL. A: EDC crosslinks identified in Fig. 7 by mass spectrometry are highlighted by yellow dashed lines between hLPL and the acidic tail of GPIHBP1^1–131^. LPL is represented by an electrostatic surface representation, whereas GPIHBP1 is shown in a cartoon representation with the sidechains of the acidic residues shown as sticks. The sidechain of Tyr^18^ is also shown by sticks, but its modification with sulfate is replaced by phosphate (a surrogate used for the AlphaFold 3 structure prediction). B: Same presentation as in (A), but with the LPL surface shown in wheat except for the surface area of residues 279–293, which is colored by atoms (carbon is white; oxygen is red, and nitrogen is blue). In an earlier HDX-MS study (18), this region was found to be transiently protected from hydrogen-deuterium exchange by the acidic tail of GPIHBP1. As the N-terminal Gln in the GPIHBP1^1–131^ preparation is almost quantitatively converted to pyro-Glu, the EDC crosslink between Gln^1^ in GPIHBP1 and Glu^289^ in LPL was only detected in GPIHBP1^Cluster1-neutral^.

With zero-length crosslinking and AlphaFold 3 modeling (Fig. 8A), we gained structural insights into why charge-cluster 2 is decisive for GPIHBP1’s ability to dislodge HSPG-bound LPL by competitive displacement. Prominent crosslinks were thus established between charge-cluster 2 in GPIHBP1’s disordered tail and lysine residues in the upper region of LPL’s C-terminal domain (i.e., Lys^445^, Lys^441^, Lys^414^, and Lys^407^). Of particular interest was the observation that Lys^445^, in the disordered C-terminal tail of LPL, interacted with an extensive charge-charge network as evidenced by its crosslinks to several acidic residues in the distal portion of charge-cluster 2 (i.e., Glu^30^, Glu^29^, Glu^27^, Glu^25^, and Glu^23^) in GPIHBP1 (Fig. 8A). We interpret these abundant crosslinks as being surrogate reporters of a high encounter probability between LPL and GPIHBP1 at these sites. In the current studies, the carboxylates in GPIHBP1 were preactivated with EDC before crosslinking was initiated by adding LPL. This sequential procedure favored the crosslinking of ensembles of Lys and Asp/Glu residues participating in the first LPL–GPIHBP1 encounters. Importantly, we found that GPIHBP1 charge-cluster 2 was indispensable for the dislodgment of HSPG-bound LPL (Fig. 6) and that cluster 2 also had the greatest EDC crosslinking efficiency to LPL (Fig. 8A). In combination, these observations suggested that the displacement of HSPG-bound LPL by GPIHBP1 resulted from a polyelectrolyte driven invasion of the LPL•HSPG binding interface. Distal acidic residues of charge-cluster 2 in the disordered tail of GPIHBP1 appeared particularly important in this process. It remains to be explored if the putative involvement of Lys^445^ in competitive LPL displacement has any bearing on the association of a gain-of-function LPL variant (LPL^S447X^) with lower plasma triglyceride levels and lower risk of ASCVD (56, 57). The importance of the electrostatic interaction between LPL and charge-cluster 2 in GPIHBP1 is illustrated by the weak and diffuse crosslinking of GPIHBP1 to LPL when this charge-cluster was neutralized (supplemental Fig. S7). It should, however, be emphasized that our crosslinking approach has an experimental bias towards lysine contributions since the side chain of arginine residues are inert to the employed EDC chemistry.

While we have shown that charge cluster 2 in the acidic tail of GPIHBP1 is essential for the competitive displacement of LPL from HSPGs, the actual sequence of the acidic residues in this cluster is likely of lesser importance than its positioning close to the heparin-binding basic patch on LPL. The crystal structure of GPIHBP1•LPL complexes shows that the acidic tail of GPIHBP1 does not fold upon LPL binding, but remains disordered forming a fuzzy complex with LPL (27). Recent studies have shown that in such polyeletrolyte interactions, neither the sequence nor the chirality of the acidic residues is decisive for the binding kinetics (58, 59) thus supporting the major importance of the positioning and the electrical field strength of the acidic tail residues for GPIHBP1-mediated competitive displacement of HSPG-bound LPL.

Our current mapping of the electrostatic interface between LPL and the acidic tail in GPIHBP1 also addresses the stabilizing effect of GPIHBP1 binding on LPL conformation. We found that the polyanionic tail of GPIHBP1 interfaced with the entirety of LPL’s cationic patch spanning both N- and C-terminal domains (Fig. 8A). Those polyelectrolyte interactions most likely stabilize LPL by reducing the flexibility of nearby structures as evidenced by their reduced deuterium uptake (Fig. 8B) and by reducing the internal electrostatic repulsion from the high cationic charge-density of the heparin-binding sites in LPL. The proposition that the inherent metastability of LPL is rooted in electrostatic repulsion within its cationic patch awaits further experimental testing.

## Data availability

All data are contained within the article and its supplements.

## Supplemental data

This article contains supplemental data (28, 60, 61).

## Conflict of interest

The authors declare that they have no conflicts of interest with the contents of this article.

## Footnotes

1 Throughout this article, sequence numbering starts with the first residues at the N-terminus of the mature protein, i.e. without the signal sequence.
