# Supplementary material for: Competitive displacement of lipoprotein lipase from heparan sulfate is orchestrated by a disordered acidic cluster in GPIHBP1
Source: J Lipid Res. 2025 Jan 13;66(2):100745. doi: 10.1016/j.jlr.2025.100745 (PMC11869522; doi:10.1016/j.jlr.2025.100745)
Supplement: Supplementary data [file mmc1.pdf]

J Lipid Research: Supplementary section

Competitive displacement of lipoprotein lipase from heparan sulfate is orchestrated by a disordered acidic cluster in GPIHBP1

Anamika Biswas[1,2], Samina Arshid[3], Kristian Kølby Kristensen[1,2], Thomas J.D. Jørgensen[3], and Michael Ploug[1,2*]

[1]Finsen Laboratory, Copenhagen University Hospital – Rigshospitalet, Copenhagen, Denmark; [2]Biotech Research and Innovation Centre, University of Copenhagen, Copenhagen, Denmark; [3]Department of Biochemistry and Molecular Biology, University of Southern Denmark, DK–5230 Odense M, Denmark.

This section contains: ***Supplementary Materials & Methods***

Supplementary Figures S1–S7

Supplementary Tables S1 and S2

Supplementary References

Materials & Methods

Native PAGE

To confirm interactions between purified human GPIHBP1$^{1–131}$, GPIHBP1$^{Cluster1\text{-}neutral}$, or GPIHBP1$^{Cluster2\text{-}neutral}$ with human, mouse, and bovine LPL, these protein pairs were incubated for 30 min on ice (4 µM LPL with 18 µM GPIHBP1) before they were analyzed by native PAGE. As these concentrations were well above the K_D of 0.2 nM for the wildtype LPL•GPIHBP1 interaction, LPL is saturated with GPIHBP1 in these experiments (1). Samples were loaded on a 4–16% native polyacrylamide gel (Novex, ThermoFisher) and electrophoresed in Tris-Glycine (pH 8.4) at 100 V for 10 min, 200 V for 30 min, and 300 V for 20 min at 4°C (submerged in wet ice). After electrophoresis, protein bands were visualized by Coomassie Brilliant Blue G-250 staining of the polyacrylamide gels.

Mass spectrometry and circular dichroism spectroscopy

The intact mass of purified GPIHBP1$^{1–131}$, GPIHBP1$^{Cluster1\text{-}neutral}$, and GPIHBP1$^{Cluster2\text{-}neutral}$ were recorded on a SynaptG2 electrospray ionization mass spectrometer (Waters). Circular dichroism (CD) spectra for GPIHBP1$^{1–131}$, GPIHBP1$^{Cluster1\text{-}neutral}$, and GPIHBP1$^{Cluster2\text{-}neutral}$ were recorded on a Jasco 1500 after buffer exchange to 10 mM phosphate and 50 mM NaCl by size exclusion chromatography using a Sephadex G75 Increase 5/150 column. CD spectra for the purified GPIHBP1 variants were recorded at 25°C in Helman cuvettes (path-length of 1 mm) with a scan rate of 50 nm/min covering the range of 190-250 nm, averaging three scans for each protein. Spectra for the proteins were compared after converting to molar ellipticity using the following equation,

$$\text{molar ellipticity (deg•cm}^2\text{•dmol}^{-1}\text{)} = \frac{Elipticity\ (mdeg) * Mw}{10 * c * l * Nr}$$

where, *Mw* is the molecular weight of the protein, *c* is the protein concentration in g/l, *l* is the path-length of the cuvette, *Nr* number of amino acid residues in the protein.

Figure legends

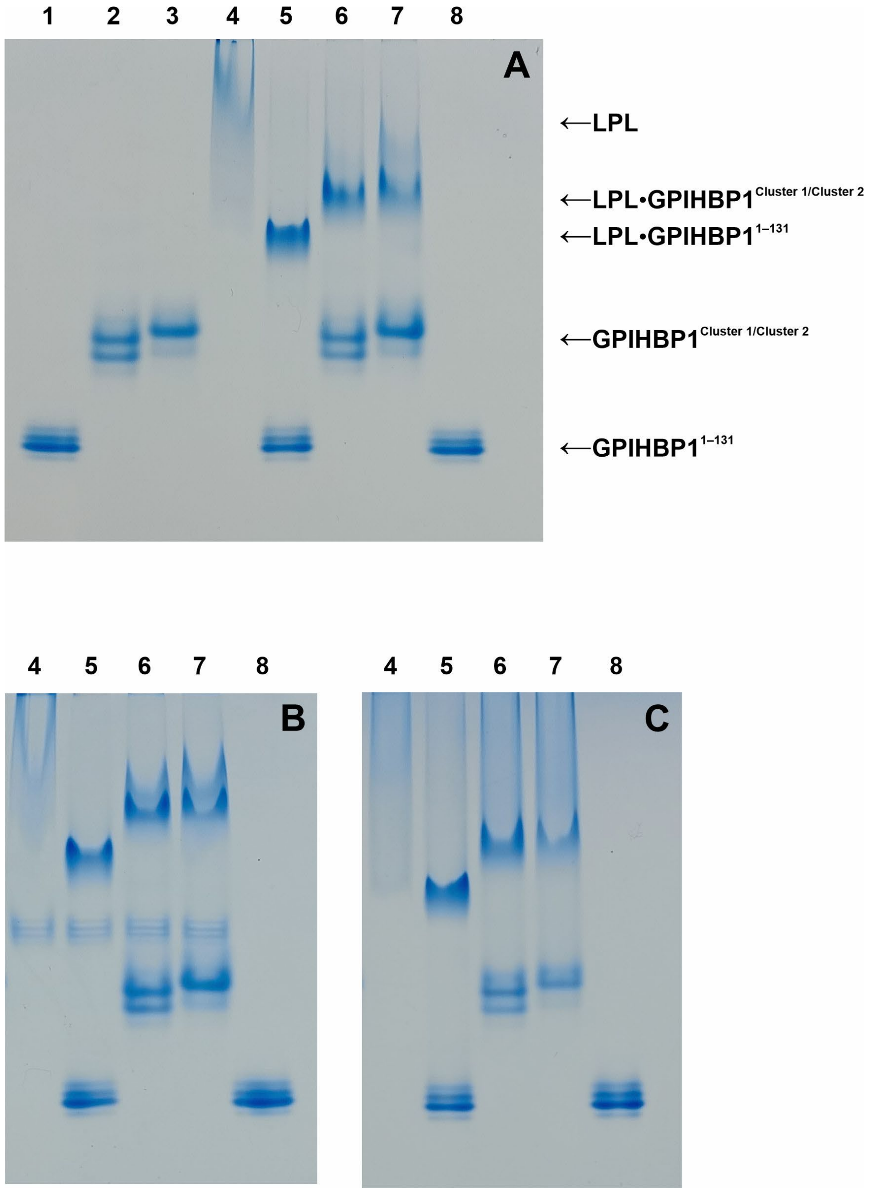

Figure S1: Binding of GPIHBP1 charge mutants to LPL as assessed by Native PAGE. The electrophoretic mobility of GPIHBP1 variants alone or in presence of purified human LPL ***(A)***, bovine LPL ***(B)***, and murine LPL ***(C)*** was assessed by native PAGE followed by Coomassie blue staining. GPIHBP1 was added in a molar excess compared to LPL. Lane 1: GPIHBP1$^{1–131}$; lane 2: GPIHBP1$^{Cluster1\text{-}neutral}$; lane 3: GPIHBP1$^{Cluster2\text{-}neutral}$; lane 4: LPL; lane 5: LPL + GPIHBP1$^{1–131}$; lane 6: LPL + GPIHBP1$^{Cluster1\text{-}neutral}$; lane 6: LPL + GPIHBP1$^{Cluster2\text{-}neutral}$; Lane 8: GPIHBP1$^{1–131}$.

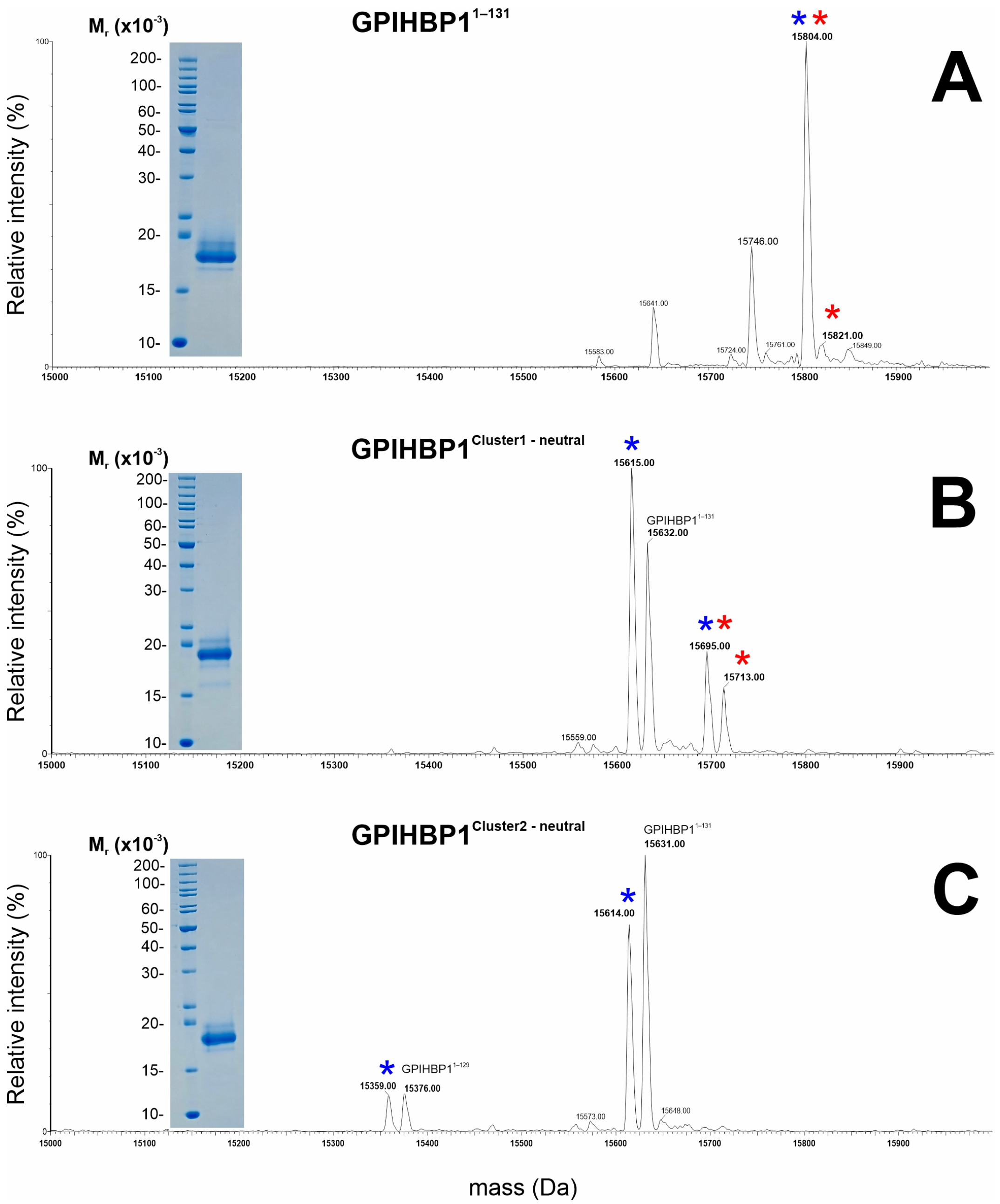

Figure S2: Characterization of purified recombinant GPIHBP1 by electrospray ionization mass spectrometry. ***(A)*** The dominant form of GPIHBP1$^{1-131}$ wild-type contained one bi-antennary N-linked carbohydrate (+1038 Da), a tyrosine sulfation on Tyr^{18} (+80 Da; *red asterisk*), and an N-terminal pyroglutamate (−17 Da; *blue asterisk*). ***(B)*** GPIHBP1$^{Cluster1\text{-}neutral}$ contained a reduced degree of sulfation on Tyr^{18} (*red asterisk*). ***(C)*** GPIHBP1$^{Cluster2\text{-}neutral}$ was not modified by tyrosine sulfation, but a small fraction was cleaved at the acid sensitive Asp^{129}–Pro^{130} bond (GPIHBP1$^{1-129}$). GPIHBP1$^{Cluster1\text{-}neutral}$ and GPIHBP1$^{Cluster2\text{-}neutral}$ have ~50% cyclization of the N-terminal glutamine to pyroglutamate (*blue asterisk*).

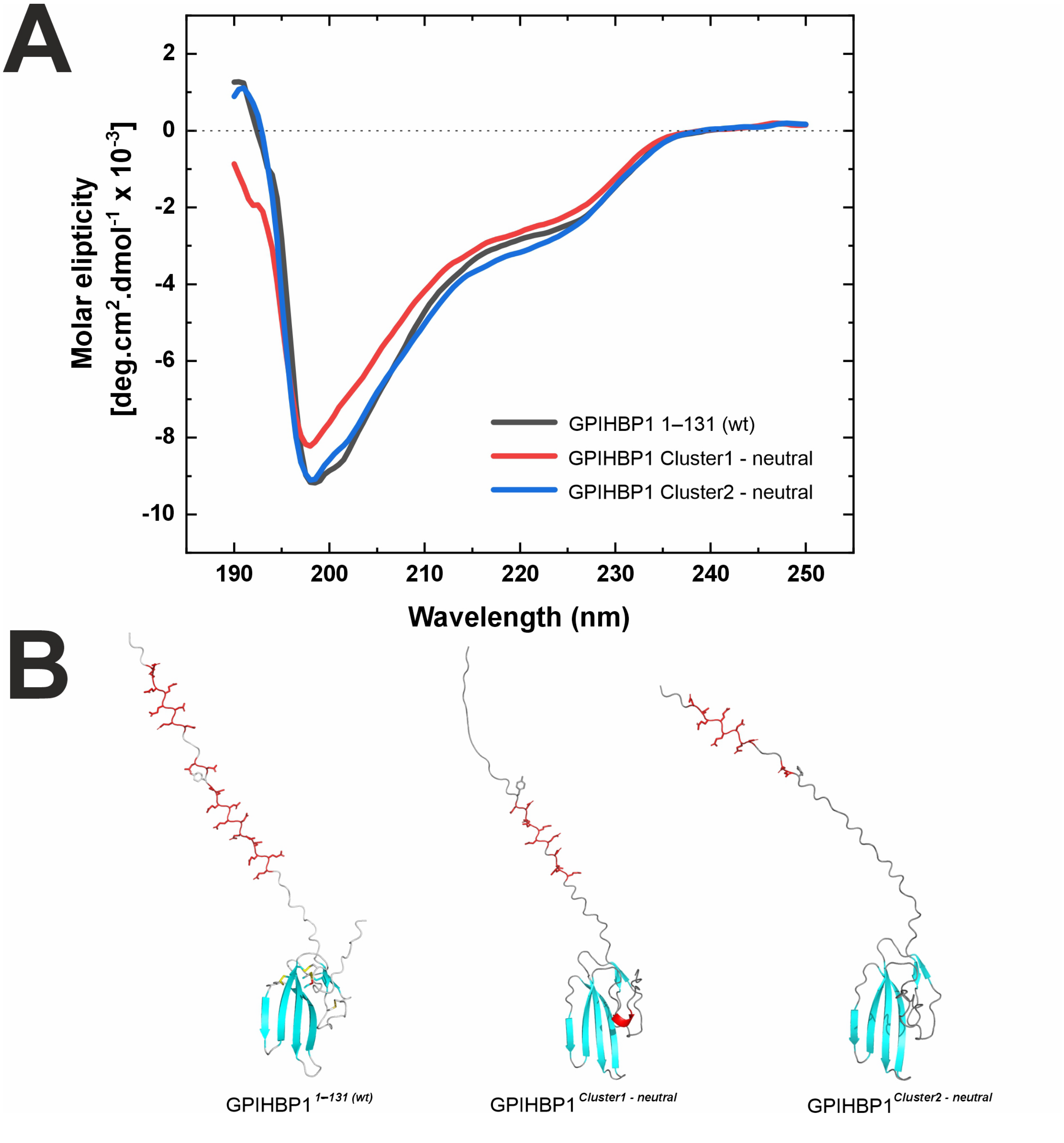

Figure S3: Secondary structures of GPIHBP1 wt and cluster 1 and 2 charge mutants. (**A**) Far-UV CD spectra recorded for GPIHBP1$^{1–131}$, GPIHBP1$^{Cluster1\text{-}neutral}$, and GPIHBP1$^{Cluster2\text{-}neutral}$ are super imposed. Estimation of the average secondary structure content using BeStSel (2) reveals an undetectable α-helix levels in all three variants, a β-sheet content of 40.3–41.3 %, turns of 17.3–18.3 %, and random coil of 41.4–42.2 %. (**B**) AlphaFold 3 does not detect any α-helix in the acidic tail of any of the GPIHBP1 mutants. Acidic residues in the disordered tail are shown as red sticks.

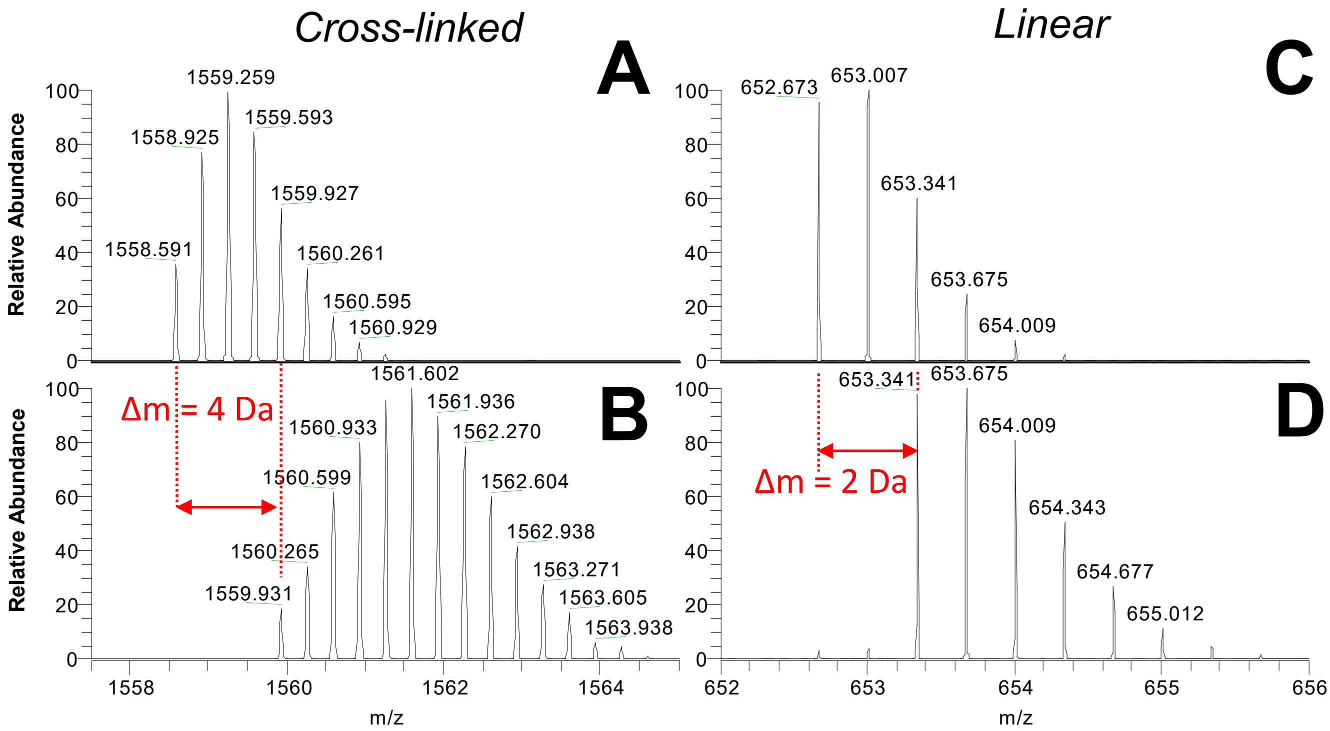

Figure S4: Verification of EDC-crosslinked peptides by their incorporation of ^{18}O after trypsin cleavage in $H_2{}^{18}O$. Isotopic envelopes of triply protonated crosslinked peptide between GPIHBP1$^{1–33}$ and hLPL$^{442–446}$, cross-linked at Glu^{25} and Lys^{445} as determined by MS/MS (Fig. S5), obtained from trypsin digestion of excised gel band with cross-linked hLPL and GPIHBP1$^{1–131}$ in (**A**) H_2O (**B**) $H_2{}^{18}O$; and the linear triply protonated LPL peptide, ^{244}SIHLFIDSLLNEENPSK^{260}, derived from trypsin digestion in (**C**) H_2O (**D**) $H_2{}^{18}O$. The cross-linked peptide exhibits a minimal mass increase of 4 Da due to the incorporation of ^{18}O atoms at two C-termini, while the linear peptide shows a minimal mass increase of 2 Da due to the incorporation of ^{18}O at a single C-terminus.

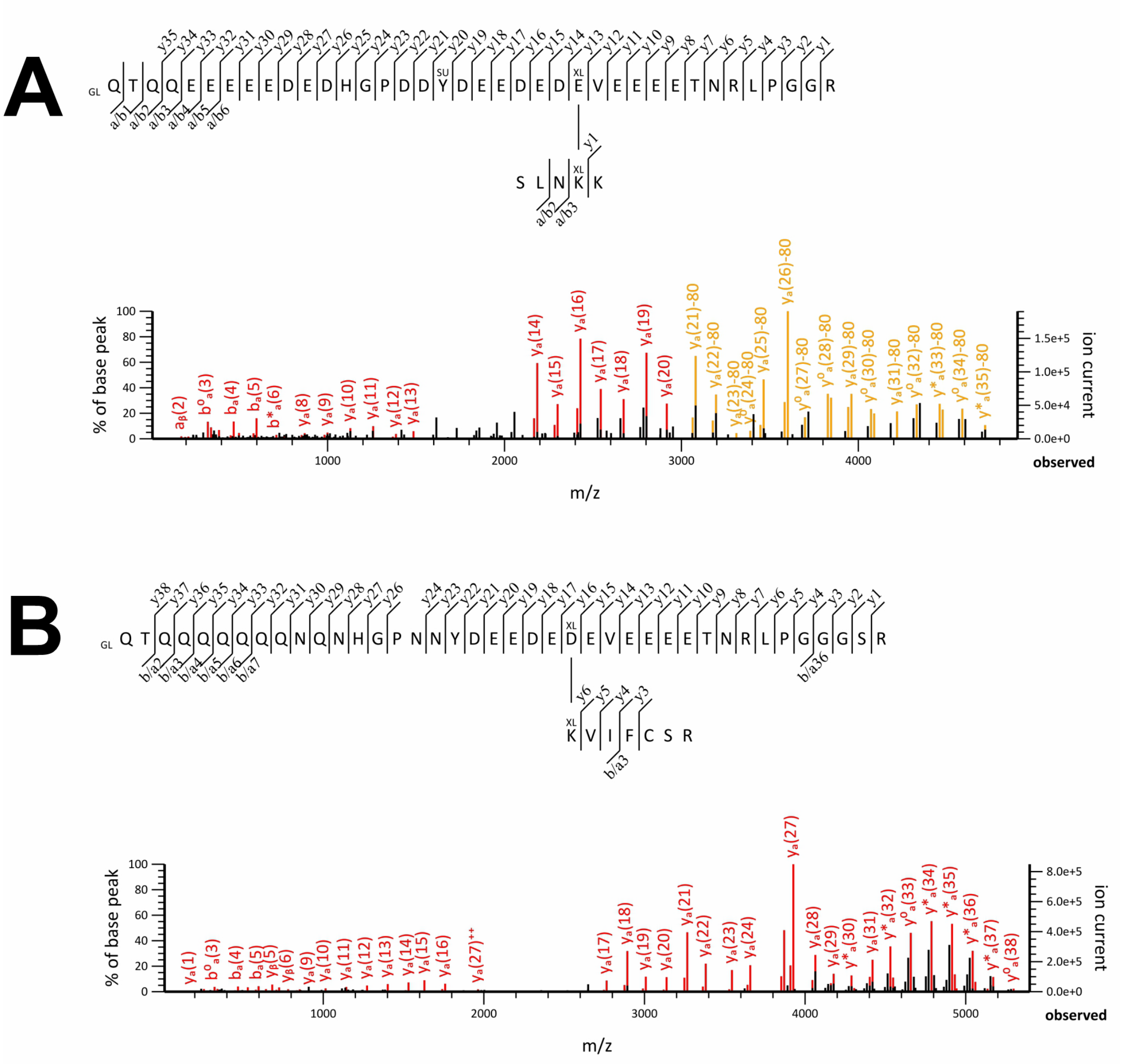

Figure S5: Identification of EDC crosslinking sites by tandem mass spectrometry (MS/MS) experiments. Representative MS/MS spectra of EDC-crosslinked hLPL and GPIHBP1$^{1–131}$ tryptic peptides. (**A**) Crosslinked peptide between GPIHBP1$^{1–38;\,wt}$ and hLPL$^{442–446}$ (monoisotopic mass of neutral linked peptide 5153.03 Da), which identifies the crosslink between GPIHBP1$^{E20;\,wt}$ and hLPL^{K445}. In wild-type GPIHBP1$^{1–131}$, Tyr^{18} was modified by sulfate, and this posttranslational modification is readily lost upon collision-induced dissociation as evidenced by the 80 Da neutral loss (-SO_3) for the y-fragment ions encompassing this residue in the tryptic GPIHBP1$^{1–38}$ fragment (SU) (**B**) Crosslinked peptide between GPIHBP1$^{1\text{-}40;\,\mathit{Cluster1\text{-}neutral}}$ and hLPL$^{414–420}$ (monoisotopic mass of neutral linked peptide 5527.42 Da), which identifies the crosslink between GPIHBP1$^{D25;\,\mathit{Cluster1\text{-}neutral}}$ and hLPL^{K414}. In both GPIHBP1$^{1–38}$ fragments, the N-terminal Gln was converted to pyro-Glu (GL). Note the jump in masses of fragment ions spanning the crosslinking site. Nomenclature of the fragment ions follows the consensus proposed by Roepstorff & Fohlman (3).

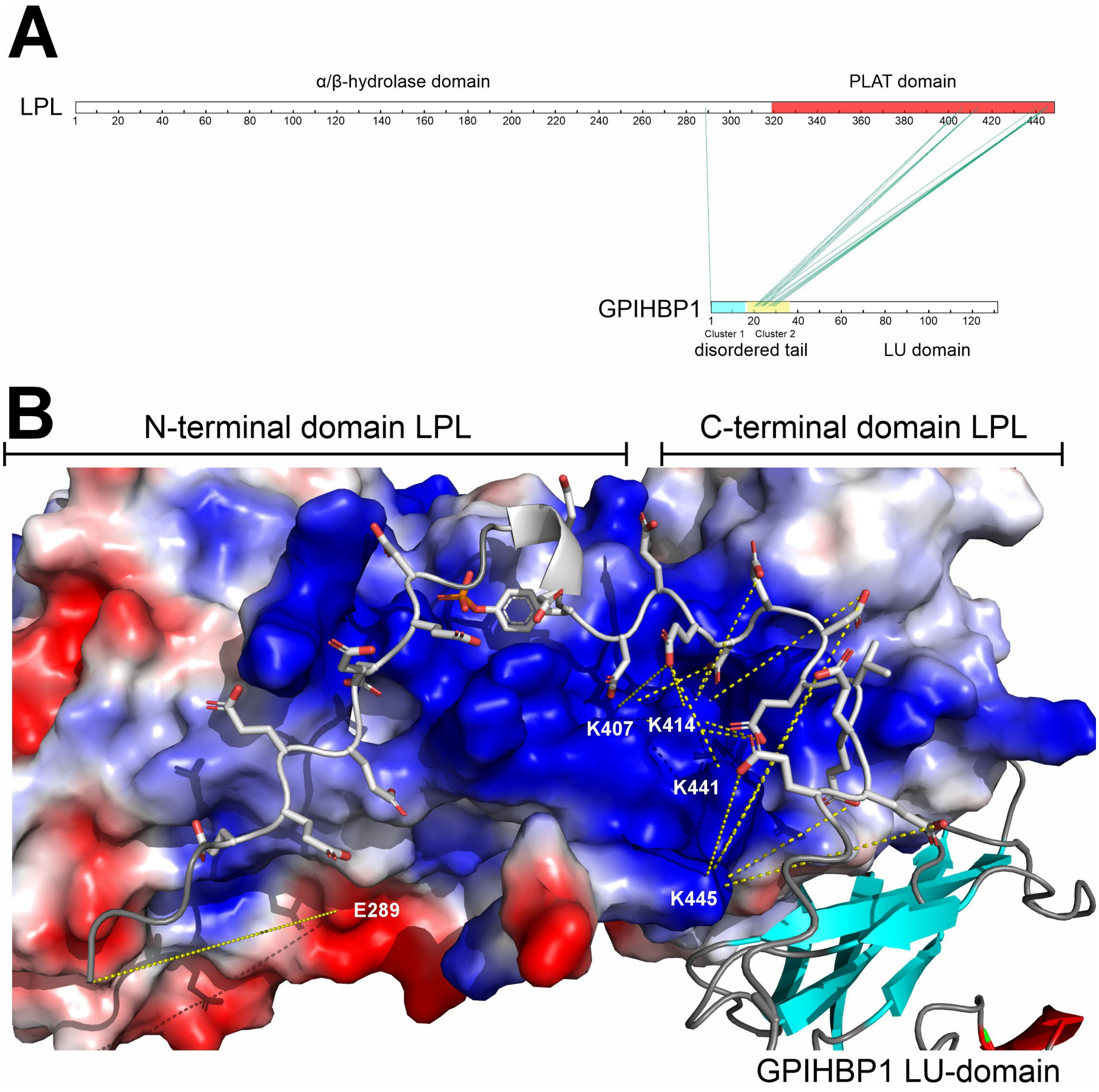

Figure S6: Crosslinks between hLPL and GPIHBP1$^{Cluster1\text{-}neutral}$. (**A**) xiVIEW representation showing the 16 EDC crosslinks determined between hLPL and GPIHBP1$^{Cluster1\text{-}neutral}$ with a Mascot ion score > 80. Internal crosslinks are omitted from this presentation. The red box highlights the C-terminal domain in LPL, while cluster1-neutral and charge-cluster2 in the disordered tail of GPIHBP1 are highlighted by cyan and yellow boxes, respectively. (**B**) Mapping these crosslinks on the model of the binding interface between the acidic intrinsically disordered tail of GPIHBP1 and the cationic surface of LPL. GPIHBP1 is shown in a cartoon representation with the acidic residues in the disordered tail shown as sticks LPL is shown in an electrostatic surface representation.

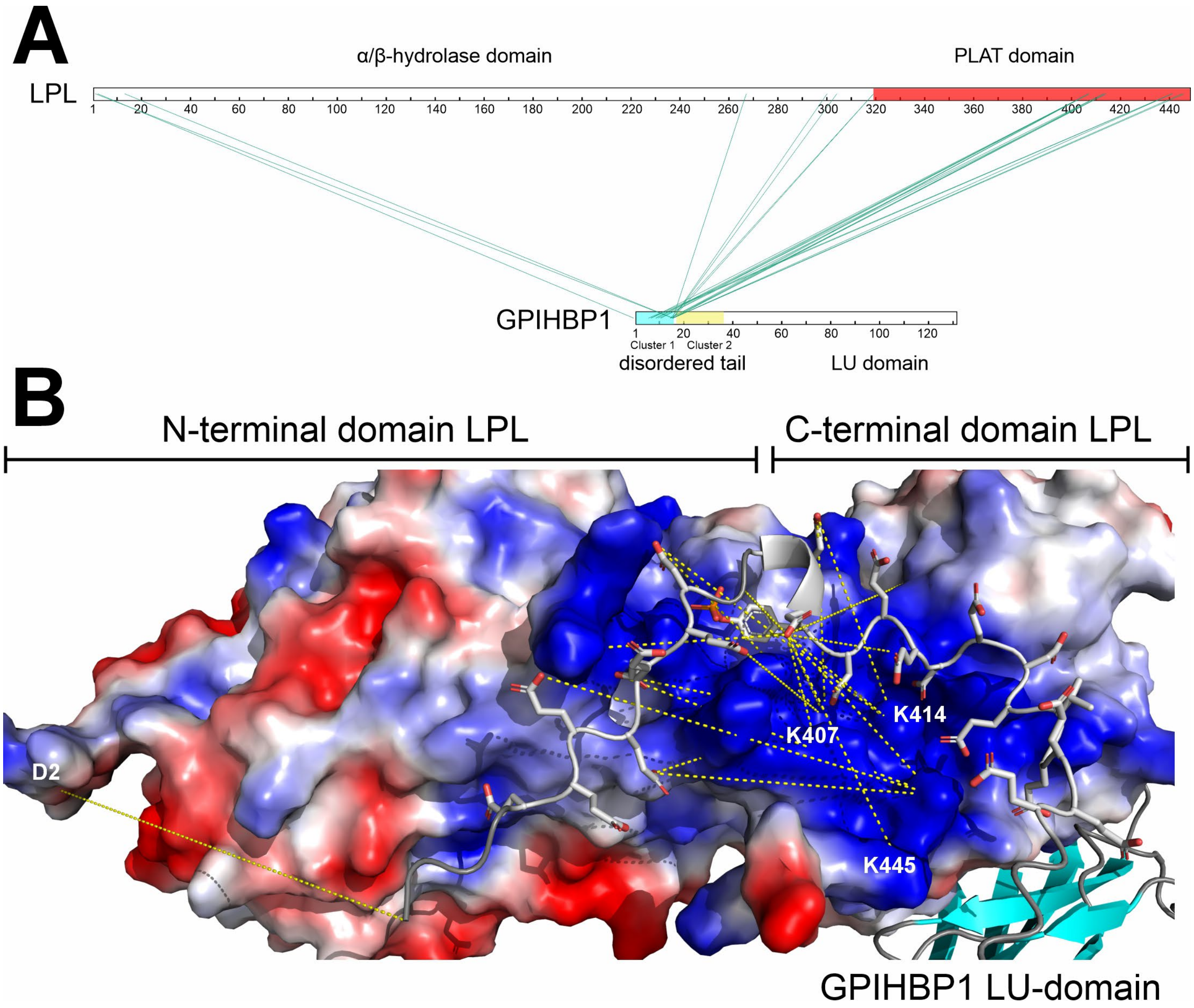

Figure S7: Crosslinks between hLPL and GPIHBP1*Cluster2-neutral*. (**A**) xiVIEW representation showing the 22 EDC crosslinks determined between hLPL and GPIHBP1*Cluster2-neutral* with a Mascot ion score > 80. Internal crosslinks are omitted from this presentation. The red box highlights the C-terminal domain in LPL, while charge-cluster1 and cluster2-neutral in the disordered tail of GPIHBP1 are highlighted by cyan and yellow boxes, respectively. (**B**) Mapping these crosslinks on the model of the binding interface between the acidic intrinsically disordered tail of GPIHBP1 and the cationic surface of LPL. GPIHBP1 is shown in a cartoon representation with the acidic residues in the disordered tail shown as sticks LPL is shown in an electrostatic surface representation.

Supplementary Tables

Table S1

	Residues	*GPIHBP1 peptides*[1]
**(1)**	*1–33*	QTQQEEEEEDEDHGPDDYDEEDEDEVEEEETNR
**(2)**	*5–33*	EEEEEDEDHGPDDYDEEDEDEVEEEETNR
**(3)**	*9–33*	EDEDHGPDDYDEEDEDEVEEEETNR
**(4)**	*13–33*	HGPDDYDEEDEDEVEEEETNR
**(5)**	*17–33*	DYDEEDEDEVEEEETNR
**(6)**	*21–33*	EDEDEVEEEETNR
**(7)**	*25–33*	EVEEEETNR
**(8)**	*1–30*	QTQQEEEEEDEDHGPDDYDEEDEDEVEEE
**(9)**	*1–25*	QTQQEEEEEDEDHGPDDYDEEDEDE
**(10)**	*1–21*	QTQQEEEEEDEDHGPDDYDEE
**(11)**	*1–17*	QTQQEEEEEDEDHGPDD
**(12)**	*1–13*	QTQQEEEEEDEDH
**(13)**	*12–21*	DHGPDDYDEE

Heparan fragments[2]	
M09 S00-biotin	IdoA–GlcNAc–IdoA–GlcNAc–IdoA–GlcNAc–IdoA–GlcNAc–Hex-*biotin*[3]
M09 S08a-biotin	IdoA–GlcNS6S–IdoA–GlcNS6S–IdoA–GlcNS6S–IdoA–GlcNS6S–Hex- *biotin*[3]

[1]Synthetic peptides covering sequences in from the intrinsically disordered N-terminal of hGPIHBP1.
[2]These defined heparan fragments were purchased from Iduron (Manchester, UK) and the codes are those used by the company. IdoA, iduronic acid; GlcNAc, N-acetyl glucosamine, GlcNS6S; *N*- and 6-*O*-sulfated glucosamine
[3]*biotin* is the product obtained after click coupling chemistry between *para*-(6-azido hexanamido)phenyl tag at reducing end of the heparin fragment and PEG4-Biotin with an alkyne group.

Table S2

MS-based identification of EDC crosslinks between hLPL and GPIHBP1

hLPL	GPIHBP1$^{1–131}$	GPIHBP1$^{Cluster1 - neutral}$	GPIHBP1$^{Cluster2 - neutral}$	Score	Match count
Lys445	Glu20			94	1
	Glu21			125	2
	Glu23			213	7
	Asp24			148	4
	Glu25			208	18
	Glu27			178	5
	Glu29			82	1
	Glu30			146	2
		Asp24		111	1
		Glu25		101	1
		Glu27		126	6
		Glu28		139	9
		Glu29		93	4
		Glu30		110	4
			Asp17	111	9
Lys441	Asp17			82	1
	Glu20			128	2
	Asp22			103	1
	Glu23			148	2
	Glu25			251	9
		Glu21		101	1
			Glu7	99	1
			Glu8	90	1
			Asp10	81	1
			Asp17	93	6
Lys414	Glu23			182	1
		Glu21		88	1
		Asp22		131	1
		Glu23		227	3
		Asp24		224	5
		Glu25		251	9
			Asp12	120	1
			Asp16	113	4
			Asp17	115	6
Lys407	Glu11			221	2
	Glu20			236	5
	Glu21			173	3
	Asp22			166	2
	Glu23			126	1
		Glu21		82	1
		Asp22		91	1
		Glu25		183	1
			Glu8	97	2
			Asp10	103	2
			Glu11	85	1
			Asp12	60	1
			Asp16	108	1
			Asp17	102	5

Ala1		Asp17	101	2
Lys13		Asp17	99	3
Lys267		Asp17	83	1
Lys300		Asp17	85	1
Lys304		Asp17	85	1
Lys319		Asp17	92	1
Lys413		Asp17	93	1
Asp2		Gln1*	94	1
Glu289	Gln1*		87	1

*Crosslinks to the α-amino group of GPIHBP1 were not detected in GPIHBP1$^{1-131}$, since this preparation contained an almost complete conversion of the α-amino group of Gln^{1} to pyroglutamic acid (**Fig. S2A**).

REFERENCES

1. Kristensen, K. K., Midtgaard, S. R., Mysling, S., Kovrov, O., Hansen, L. B., Skar-Gislinge, N. *et al.* (2018) A disordered acidic domain in GPIHBP1 harboring a sulfated tyrosine regulates lipoprotein lipase Proc Natl Acad Sci U S A **115**, E6020-E6029 10.1073/pnas.1806774115
2. Micsonai, A., Moussong, E., Wien, F., Boros, E., Vadaszi, H., Murvai, N. *et al.* (2022) BeStSel: webserver for secondary structure and fold prediction for protein CD spectroscopy Nucleic Acids Res **50**, W90-W98 10.1093/nar/gkac345
3. Roepstorff, P., and Fohlman, J. (1984) Proposal for a common nomenclature for sequence ions in mass spectra of peptides Biomed Mass Spectrom **11**, 601 10.1002/bms.1200111109
